# DELLA activity is required for successful pollen development in the Columbia ecotype of Arabidopsis

**DOI:** 10.1111/nph.12571

**Published:** 2013-11-01

**Authors:** Andrew R G Plackett, Alison C Ferguson, Stephen J Powers, Aakriti Wanchoo-Kohli, Andrew L Phillips, Zoe A Wilson, Peter Hedden, Stephen G Thomas

**Affiliations:** 1 Plant Biology and Crop Science Department, Rothamsted ResearchHarpenden, Hertfordshire, AL5 2JQ, UK; 2 School of Biosciences, University of Nottingham, Sutton Bonington CampusLoughborough, Leicestershire, LE12 5RD, UK; 3 Biomathematics and Bioinformatics Department, Rothamsted ResearchHarpenden, Hertfordshire, AL5 2JQ, UK

**Keywords:** *Arabidopsis thaliana*, Col-0, DELLA, ecotypic differences, gibberellin, L*er*, male sterility, pollen development

## Abstract

- Excessive gibberellin (GA) signalling, mediated through the DELLA proteins, has a negative impact on plant fertility. Loss of DELLA activity in the monocot rice (*Oryza sativa*) causes complete male sterility, but not in the dicot model Arabidopsis (*Arabidopsis thaliana*) ecotype Landsberg *erecta* (L*er*), in which DELLA function has been studied most extensively, leading to the assumption that DELLA activity is not essential for Arabidopsis pollen development. A novel DELLA fertility phenotype was identified in the Columbia (Col-0) ecotype that necessitates re-evaluation of the general conclusions drawn from L*er*.
- Fertility phenotypes were compared between the Col-0 and L*er* ecotypes under conditions of chemical and genetic GA overdose, including mutants in both ecotypes lacking the DELLA paralogues *REPRESSOR OF ga1-3* (*RGA*) and *GA INSENSITIVE* (*GAI*).
- L*er* displays a less severe fertility phenotype than Col-0 under GA treatment. Col-0 *rga gai* mutants, in contrast with the equivalent L*er* phenotype, were entirely male sterile, caused by post-meiotic defects in pollen development, which were rescued by the reintroduction of DELLA into either the tapetum or developing pollen.
- We conclude that DELLA activity is essential for Arabidopsis pollen development. Differences between the fertility responses of Col-0 and L*er* might be caused by differences in downstream signalling pathways or altered DELLA expression.

## Introduction

The phytohormone gibberellin (GA) regulates stamen development in numerous flowering plants (Pharis & King, 1985). GA is necessary for filament elongation and pollen development in Arabidopsis (*Arabidopsis thaliana*) and rice (*Oryza sativa*) (reviewed in Plackett *et al*., 2011): anther development arrests prematurely in mutants unable to synthesize or perceive bioactive GA in these two species and tomato (Nester & Zeevaart, 1988; Jacobsen & Olszewski, 1991; Goto & Pharis, 1999; Cheng *et al*., 2004; Aya *et al*., 2009). Expression analyses suggest that GA signalling occurs in the anther tapetum and developing microspores (Hirano *et al*., 2008; Hu *et al*., 2008). GA signalling in rice anthers acts through the transcription factor OsGAMYB (Aya *et al*., 2009), with downstream targets regulating tapetum secretory functions and programmed cell death (PCD). Two GAMYB homologues in Arabidopsis, MYB33 and MYB65, have similar functions (Millar & Gubler, 2005), suggesting that GA regulation of stamen development is conserved between monocots and dicots.

GA signalling acts through the degradation of DELLA proteins (reviewed in Harberd *et al*., 2009; Ueguchi-Tanaka & Matsuoka, 2010; Sun, 2010), a class of transcriptional regulators belonging to the GRAS (for GA INSENSITIVE (GAI), REPRESSOR OF *ga1-3* (RGA) and SCARECROW (SCR)) superfamily (Pysh *et al*., 1999) that otherwise inhibit GA-dependent changes in the expression of downstream target genes (Cao *et al*., 2006; Zentella *et al*., 2007; Hou *et al*., 2008). DELLA loss-of-function mutants display constitutive growth responses which mimic treatment with exogenous GA (Silverstone *et al*., 1997; Ikeda *et al*., 2001). DELLA-dependent transcriptional regulation is mediated through protein–protein interactions with multiple classes of transcription factors, the first described example of this being the sequestration of PHYTOCHROME INTERACTING FACTOR (PIF)3 and PIF4 to inhibit hypocotyl elongation during photomorphogenesis (de Lucas *et al*., 2008; Feng *et al*., 2008).

Although most recent investigations have concentrated on the effects of GA deficiency or insensitivity on stamen development, excessive GA signalling also negatively affects Arabidopsis fertility. Wild-type Columbia (Col-0) and GA biosynthetic mutants set fewer seeds per silique when grown under exogenous GA treatment (Jacobsen & Olszewski, 1993; Rieu *et al*., 2008a,b). Arabidopsis possesses five DELLA paralogues (Dill & Sun, 2001), and studies on their function have been conducted mainly in Landsberg *erecta* (L*er*). It has been shown recently that a basal level of fertility persists in the L*er* ecotype, even in the *global* mutant, which carries mutations in all five DELLA paralogues (Fuentes *et al*., 2012). By contrast, loss-of-function mutants in the monocot species rice and barley (*Hordeum vulgare*), which each possess single DELLA orthologues, are reported as sterile (Lanahan & Ho, 1988; Ikeda *et al*., 2001).

In this article, we report the creation of a novel *rga gai* loss-of-function mutant in the Col-0 ecotype, *rga-28 gai-td1*, which display complete male sterility, contrary to the phenotype of L*er* DELLA mutants. Plants in which DELLA activity was not impaired did not display such severe fertility phenotypes when grown under exogenous GA treatment. Loss of ERECTA (ER) function from the Col-0 DELLA loss-of-function mutant *rga-28 gai-td1* did not restore fertility, and so cannot explain the phenotypic differences between Col-0 and L*er* DELLA mutants. These findings highlight hitherto unsuspected ecotypic differences in floral developmental responses to GA, and imply potential differences in GA signal transduction or downstream transcriptional networks between Col-0 and L*er*.

Microscopic analysis of *rga-28 gai-td1* anthers identified post-meiotic developmental defects leading to the collapse of immature pollen. Reintroduction of RGA rescued male fertility, confirming the necessity of DELLA activity to pollen development in Col-0. Surprisingly, it was found that the expression of RGA separately in either the tapetum or developing microspores was sufficient to restore pollen development, raising the prospect of post-meiotic communication between these two distinct and physically separate cell lineages downstream of the DELLA proteins.

## Materials and Methods

### Plant material and growth conditions

Experiments were performed in either the *Arabidopsis thaliana* (L.) Heynh. Col-0 or L*er* ecotype, as specified. Plant growth conditions and exogenous 100 µM GA_3_ treatment were as described in Rieu *et al*. (2008b). The *rga-28* allele has been described previously in Tyler *et al*. (2004). *rga-30* was identified from the SALK collection (SALK_137951) (Alonso *et al*., 2003), *gai-td1* from the SAIL collection (SAIL_82_F06) (Sessions *et al*., 2002), and *gai-td2* (SK14663) and *gai-td3* (SK20878) from the SK population of activation tagged lines (Robinson *et al*., 2009) (Supporting Information Fig. S1). The *rga-24*,*rga-t2* and *gai-t6* alleles have been described previously (Peng *et al*., 1997; Dill & Sun, 2001; Lee *et al*., 2002). *rga-28 gai-td1*,*rga-28 gai-td2*,*rga-28 gai-td3*,*rga-30 gai-td1*,*rga-30 gai-td2* and *rga-30 gai-td3* double-mutant lines were established by crossing and were verified by genotyping PCR (Table S1).

### Transgenic lines

The *RGA* CDS lacking a stop codon was cloned into pENTR11 as a *Sal*I-*Not*I restriction fragment and subsequently fused in frame with green fluorescent protein (GFP) in the pGWB450 vector (Nakagawa *et al*., 2007) via Gateway LR recombination (Invitrogen, Carlsbad, CA, USA).

Published fragments of the *LTP12* (Ariizumi *et al*., 2002) or *LAT52* (Twell *et al*., 1989) promoter sequence were introduced as *Xba*I restriction fragments. Segregating *rga-28 gai-td1/+* populations were transformed via *Agrobacterium*, with subsequent selection of T_1_ transformants by kanamycin resistance. T_1_ individuals homozygous for both *rga-28* and *gai-td1* were identified by genotyping PCR (Table S1). Single insertion transgenic lines descended from these individuals were identified in the T_2_ generation using 3 : 1 Mendelian segregation, with the presence of the transgene confirmed by PCR.

### Phenotypic characterization

Growth characterization experiments were performed using a blocked split plot design (Gomez & Gomez, 1984) with GA treatment applied across whole trays (main plot) and genotypes being randomized within trays (split plot). Phenotypic measurements were performed as described in Rieu *et al*. (2008b), using a population of 144 plants (*n *=* *12). Three siliques were harvested from the primary inflorescence of each plant for seed counts. The fertility of individual floral positions was scored on the basis of silique elongation by the end of flowering.

### Microscopy

Pollen viability was assessed using Alexander cytoplasmic stain (Alexander, 1969) on whole anther squashes; pollen developmental progression was determined by staining isolated stamens using 2.0 μg µl^−1^ 4′,6-diamidino-2-phenylindole (DAPI, Sigma) in aqueous solution (Tarnowski *et al*., 1991) and observed using a UV light microscope (Nikon, Tokyo, Japan). Embedding of inflorescence tissues for sectioning was performed as described in Vizcay-Barrena & Wilson (2006). Two-micrometre-thick sections were observed with a Zeiss Axiophot light microscope (Carl Zeiss Ltd, Welwyn Garden City, UK) with an attached Retiga EXi camera system (QImaging, Surrey, BC, Canada). Paclobutrazol (PAC)-treated inflorescences (1 mM PAC, 0.06% Tween-20) were used in confocal microscopy of transgenic lines to enhance DELLA stabilization and thus GFP intensity. Fluorescence was detected using a Leica SP2 confocal laser scanning microscope (Leica Microsystems, Wetzlar, Germany), exciting fluorescence at 488 nm with an argon laser. GFP excitation was collected between 500 and 515 nm, and chlorophyll excitation was collected between 660 and 700 nm. Images were processed using Leica SP2 Image Analysis software.

### Statistical analysis

All statistical analysis was performed using the Genstat software package (2010, 13th edition, ©VSN International Ltd, Hemel Hempstead, UK). Phenotypic characters were analysed by analysis of variance (ANOVA), using a transformed scale where necessary (natural logarithm for vegetative internode and silique lengths), to meet the assumptions of a normal distribution and homogeneity of variance, and taking into account the blocked split plot design. The main effects of genotype and GA treatment and the interaction between these factors were assessed by *F* tests. GA treatment did not have a significant effect on flowering time (*P *=* *0.096), but genotype was strongly significant (*P *<* *0.001). For all other characters, a significant interaction (*P *<* *0.001) was detected between genotype and GA treatment. The binary character of silique set was analysed by modelling the proportion of plants (*n *=* *12) for each genotype by GA treatment combination having set a silique at each inflorescence position, using a generalized linear model (GLM) (McCullagh & Nelder, 1989) with a logit link function. Following ANOVA or GLM, comparisons were made between pairs of genotypes or GA treatment conditions within a single genotype using least significant differences (LSDs) based on the appropriate degrees of freedom (df) with a significance threshold of either 5% or 1%, as specified. Means (in figures) are otherwise presented with individual standard errors (SE).

## Results

### Loss of functional RGA and GAI causes complete sterility in the Col-0 ecotype, but not in L*er*

**Figure 1:**
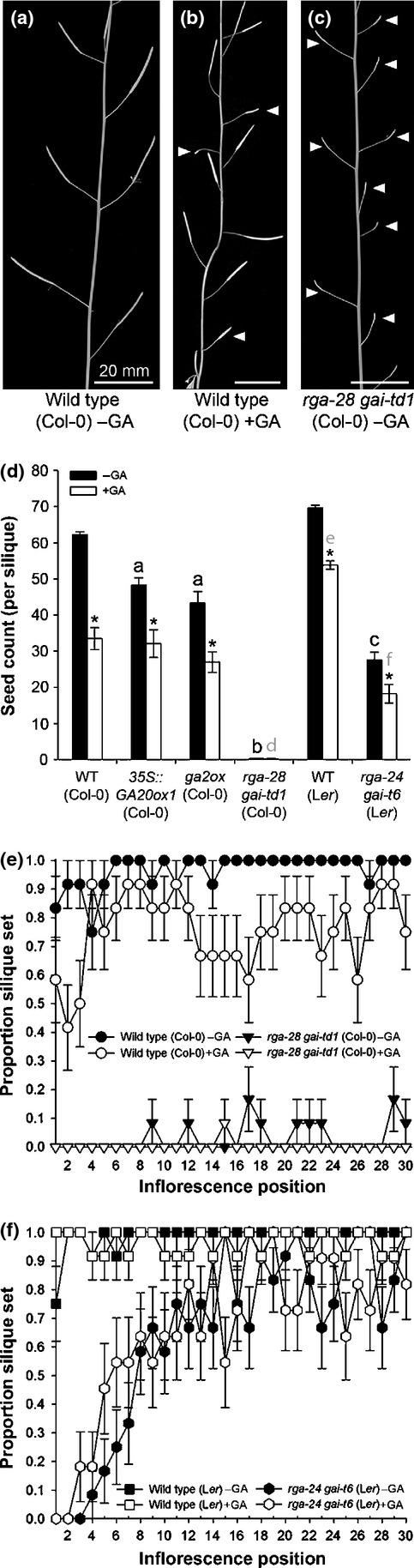
Differential fertility of *Arabidopsis thaliana* Col-0 and L*er rga gai* mutants. (a–c) Primary inflorescences showing silique set of wild-type Col-0 under (a) control growth conditions and (b) 100 µM GA_3_ treatment, and (c) *rga-28 gai-td1* (Col-0). White arrowheads indicate infertile silique positions. (d) Mean number of seeds per silique (*n *=* *36, ± SE) under control conditions and GA_3_ treatment. Letters indicate significant difference (*P *<* *0.01) of a genotype within a single GA treatment from control wild-type (black) and GA-treated wild-type (grey), respectively. Genotypes marked with different letters are significantly different from one another. Asterisks indicate significant difference (*P *<* *0.01) between GA treatments within the same genotype. Pairwise comparisons were made using 1% least significant differences (LSDs) on a log-transformed scale (8.782 on 121 df between genotypes, 9.717 on 71 df between GA treatments). (e, f) Mean frequency of successful silique set on the primary inflorescence (*n *=* *12, ± SE) of wild-type and *rga gai* mutant genotypes in Col-0 (e) and L*er* (f) under control growth conditions and GA treatment.

Exogenous GA treatment is known to reduce seed set (per silique) in the Arabidopsis Col-0 ecotype (Jacobsen & Olszewski, 1993; Rieu *et al*., 2008a,b). In addition, we found that GA treatment reduces the frequency of silique set in Col-0 (Fig.1a,b), although this phenomenon was unpredictable, with flowers immediately adjacent to infertile positions often setting fully fertile siliques (Fig.1b). Unexpectedly, a novel DELLA loss-of-function double mutant generated in the Col-0 background, *rga-28 gai-td1*, was completely infertile under control growth conditions (Fig.1c). The equivalent mutant in the L*er* ecotype, *rga-24 gai-t6*, has been shown previously to be fertile, albeit at reduced levels (Dill & Sun, 2001). The association of this phenotype with the loss of both RGA and GAI in the Col-0 background was confirmed by combining other *rga* and *gai* loss-of-function alleles. All combinations proved infertile (Fig. S1).

To further investigate the interaction between GA overdose, ecotype and fertility, lines with reduced DELLA signalling (*rga-28 gai-td1* and the comparable L*er* mutant, *rga-24 gai-t6*; Dill & Sun, 2001) or altered GA biosynthesis (*35S::GA20ox1* (Col-0) and the Col-0 *ga2ox* quintuple loss-of-function mutant, herein referred to as *ga2ox*; Coles *et al*., 1999; Rieu *et al*., 2008a) were characterized under both control growth conditions and GA treatment. Increasing bioactive GA through genetic manipulation reduced seed set (*P *<* *0.01; Fig.1d), but neither this nor further exogenous GA treatment reduced seed set to the extent displayed by either *rga gai* mutant (*P *<* *0.01). Further reductions in seed set in the two genetically GA-overdosed lines when under GA treatment (*P *<* *0.01) suggest that GA responses in these lines are not fully saturated. *rga-24 gai-t6* (L*er*) was confirmed as partially fertile under our growth conditions compared with the wild-type (*P *<* *0.01; Fig.1d). Seed set in this mutant was further reduced by GA treatment (*P *<* *0.01), but still remained more fertile than *rga-28 gai-td1* (Col-0; *P *<* *0.01). The remaining capacity of the *rga-24 gai-t6* (L*er*) reproductive tissues to respond to GA could be explained by the presence of at least one additional DELLA paralogue in this ecotype.

Interestingly, although seed number was not significantly different between Col-0 and L*er* wild-type under control growth conditions (*P *>* *0.05; Fig.1d), L*er* seed set under GA treatment was far more robust, being reduced to 77.30% of the untreated wild-type compared with 53.69% in Col-0 (*P *<* *0.01), despite indications from the similarity in seed set between GA-treated Col-0 wild-type, *35S::GA20ox1* and *ga2ox* (*P *>* *0.05) that GA treatment was sufficiently strong to saturate downstream responses. Similar conclusions can be drawn when considering the frequency of silique set across the primary inflorescence: GA treatment reduced the mean probability of silique set in Col-0 to 0.76 ± 0.02 compared with a control value of 0.97 ± 0.01 (Fig.1e), whereas the fertility of L*er* was unaffected (0.96 ± 0.01 compared with 0.99 ± 0.01; Fig.1f). Genetic GA overdose induced similar reductions in Col-0 silique set to GA treatment (Fig. S2), none of which were as severe as the infertile *rga-28 gai-td1* (Col-0) phenotype (Fig.1e). Rare *rga-28 gai-td1* (Col-0) siliques contained single seeds that probably resulted from uncontrolled cross-pollination. By contrast, early infertility of *rga-24 gai-t6* (L*er*) quickly recovered (Fig.1f) to a mean silique set frequency of 0.83 ± 0.03 from the 12th flower position onward. GA treatment had no discernible effect on fertility in this L*er* mutant. Other differential growth responses between Col-0 and L*er* were identified in a broader phenotypic characterization (Table S2), most notably the number and elongation of vegetative internodes, with the loss of RGA and GAI affecting each ecotype differently and each *rga gai* mutant responding differently to GA treatment. This includes some phenotypic differences remaining under GA treatment.

Greater loss of fertility in *rga gai* mutants compared with that when manipulating bioactive GA content might reflect different underlying causes of infertility. Reduced fertility of two GA biosynthesis mutants, *ga20ox1 ga20ox2* (Rieu *et al*., 2008b) and *ga3ox1 ga3ox3* (Hu *et al*., 2008), has been attributed to mismatched growth between the stamens and pistil, creating a mechanical block to pollination. However, non-linear modelling of Col-0 floral organ growth during flower opening (Fig. S2, Table S3, Methods S1), comparing control growth conditions and GA treatment, found no effect on stamen length relative to the pistil (over 100% at flower opening), nor on the timing of anthesis (*P *>* *0.05; 5.45 ± 0.91 h and 4.47 ± 1.37 h before flower opening under control and GA-treated conditions, respectively). Therefore, reduced fertility under GA treatment cannot be explained by a mechanical barrier to pollination.

It was noticed that anthers from GA-treated flowers appeared to be smaller than those in untreated flowers. Modelling Col-0 anther length (taken as a linear measurement from the anther–filament junction to the anther tip) in the same experimental population found that GA treatment caused a consistent significant reduction in anther size over the developmental period measured (*P *<* *0.05; Fig. S2, Table S3), suggesting that GA treatment has a direct negative effect on anther development.

### Sterility in *rga-28 gai-td1* is caused by pollen lethality

**Figure 2:**
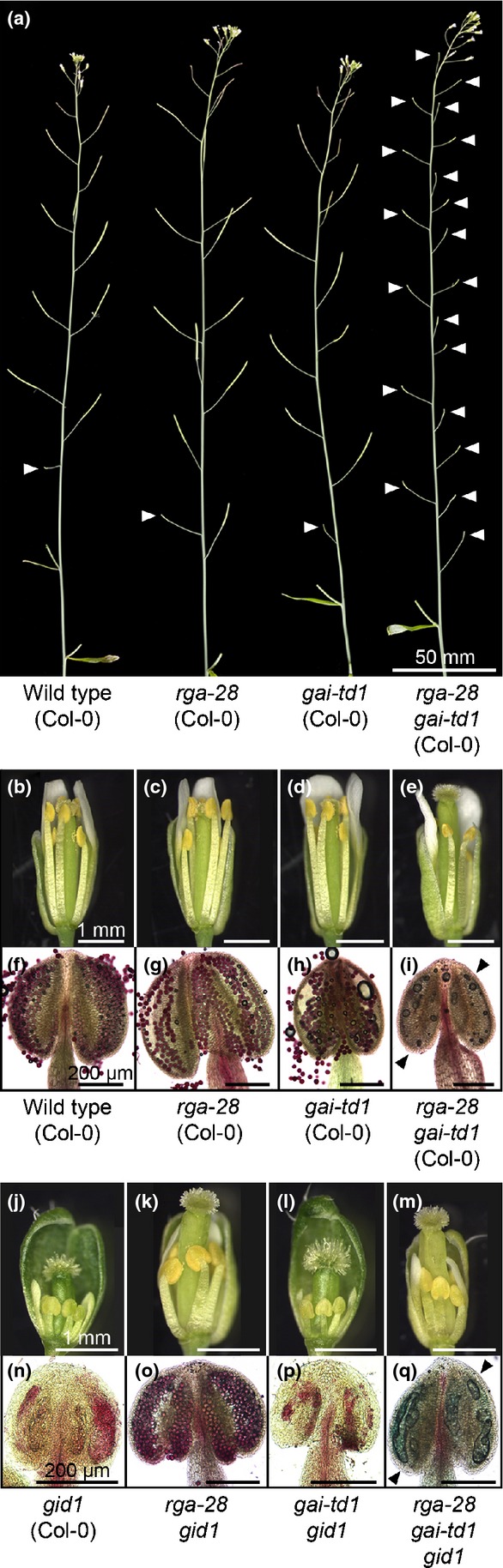
*rga-28 gai-td1* (*Arabidopsis thaliana* Col-0) is male sterile. (a) Primary inflorescence phenotypes of *rga-28 gai-td1* combinatorial mutant lines (as specified). White arrowheads indicate infertile silique positions. (b–i) Floral phenotypes of the mutant lines in (a), showing newly opened flowers (b–e) and whole anthers (f–i). Anthers have been stained to determine pollen viability (see the Materials and Methods section): dark red colouring indicates viable pollen. Black arrowheads indicate empty locules. (j–q) Floral phenotypes of *rga-28 gai-td1* combinatorial mutant lines in the *gid1* GA-insensitive mutant background (as specified), showing newly opened flowers (j–m) and whole anthers (n–q). Anthers have been stained to determine pollen viability: dark red colouring indicates viable pollen. Black arrowheads indicate empty locules.

Loss of RGA or GAI separately did not affect silique set when compared with wild-type Col-0 (Fig.2a), indicating that these paralogues act redundantly to promote fertility. In contrast with wild-type Col-0, *rga-28* and *gai-td1* flowers (Fig.2b–d, f–h), *rga-28 gai-td1* double-mutant flowers exhibited a pollenless phenotype (Fig.2e,i). *rga-28 gai-td1* flowers set siliques when pollinated manually with wild-type Col-0 pollen, indicating that infertility of *rga-28 gai-td1* (Col-0) inflorescences is caused by male sterility. The same pollenless phenotype was observed when *rga-28 gai-td1* was recapitulated in the *gid1a-1 gid1b-1 gid1c-1* (Col-0) triple GA receptor mutant background (Griffiths *et al*., 2006; herein referred to as *gid1*; Fig.2m,q). Insensitivity of *gid1* to GA signalling causes constitutive inhibition of GA responses by DELLA proteins, and thus the pollenless phenotype is necessarily dependent on downstream DELLA signalling. Infertility of *gid1* stamens caused by arrested floral development (Fig.2j,n; Griffiths *et al*., 2006) was overcome by loss of RGA alone (Fig.2k,o), but not GAI (Fig.2l,p). Pistil, stamen and petal size were all reduced in *rga-28 gai-td1 gid1* compared with *rga-28 gai-td1* (Fig.2e,m), suggesting that other DELLA paralogues repress aspects of Arabidopsis floral growth and development. A separate *gid1* triple mutant, *gid1a-1 gid1b-1 gid1c-2*, has been reported previously as non-flowering (Willige *et al*., 2007; Ragni *et al*., 2011), but, under our growth conditions, this mutant flowered at a similar time to *gid1a-1 gid1b-1 gid1c-1* and displayed a very similar floral phenotype (Fig. S3).

### Pollen development is negatively affected in *rga gai* mutants in both Col-0 and L*er*

**Figure 3:**
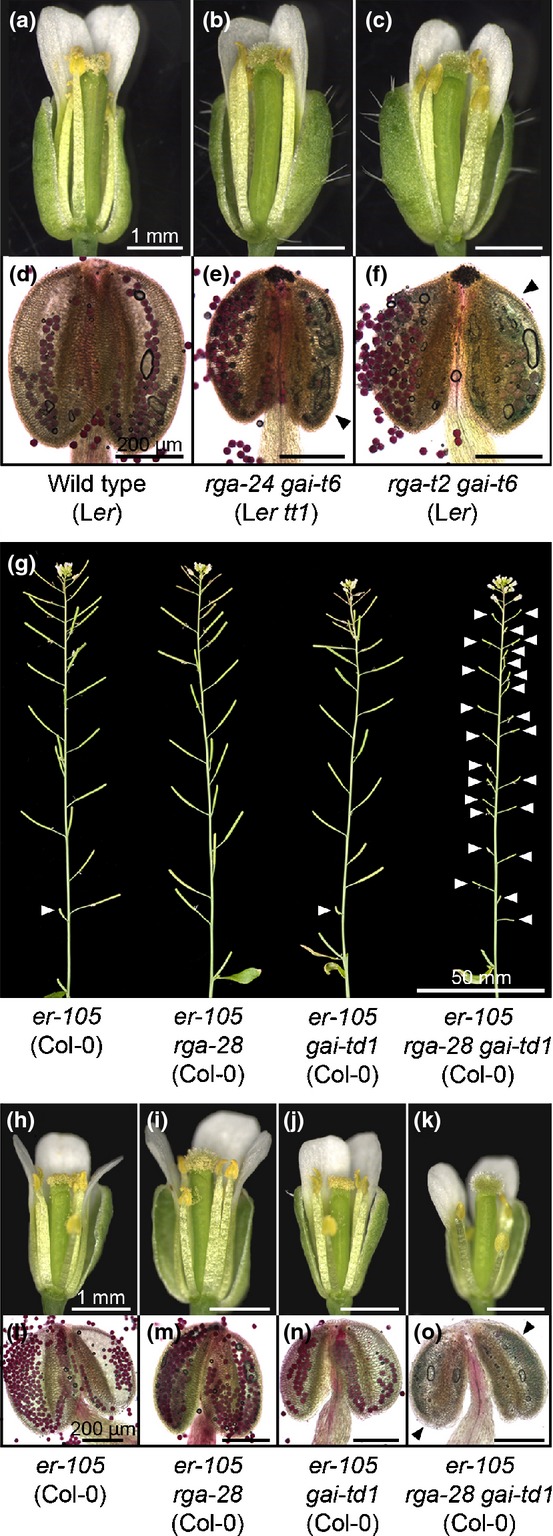
*rga gai* (*Arabidopsis thaliana* L*er*) mutants retain male fertility. (a–f) Floral phenotypes of wild-type L*er*,*rga-24 gai-t6* and *rga-t2 gai-t6*, showing newly opened flowers (a–c) and whole anthers (d–f). Anthers have been stained to determine pollen viability: dark red colouring indicates viable pollen. Black arrowheads indicate empty locules. (g) Primary inflorescence phenotypes of *er-105 rga-28 gai-td1* combinatorial mutant lines. White arrowheads indicate infertile silique positions. (h–o) Floral phenotypes of mutants described in (g), showing newly opened flowers (h–k) and whole anthers (l–o). Anthers have been stained to determine pollen viability: dark red colouring indicates viable pollen. Black arrowheads indicate empty locules.

The partial fertility of L*er rga gai* mutants (Fig.1d,f) was also investigated, examining the floral phenotypes of *rga-24 gai-t6* (which also carries *transparent testa 1*,*tt1*) and the independent line *rga-t2 gai-t6*. The stigmas of newly opened mutant flowers bore visibly less pollen than wild-type L*er* (Fig.3a–c), consistent with previous observations by Dill & Sun (2001). Under microscopic examination, individual empty locules were identified in *rga-24 gai-t6* and *rga-t2 gai-t6* anthers (Fig.3e,f). This indicates that pollen development is disrupted by the loss of RGA and GAI function in both ecotypes, but with a less severe impact on L*er* male fertility. The expression of additional DELLA paralogues in L*er* anther tissues could potentially explain this (see the Discussion section). However, the *global* DELLA (L*er*) quintuple mutant, which is reported to lack functional DELLA protein (Feng *et al*., 2008), produces viable pollen and displays a similar degree of fertility to *rga-24 gai-t6* (Fig. S4; Fuentes *et al*., 2012), suggesting that the differences between Col-0 and L*er* regulating pollen development lie downstream of the DELLAs.

Col-0 and L*er* demonstrate distinct growth habits, including striking differences in floral cluster architecture (Fig. S5), attributed, in part, to loss of the ER leucine-rich repeat receptor-like kinase (LRR-RLK) in L*er* (Torii *et al*., 1996). ER promotes stamen development in conjunction with ERECTA-LIKE (ERL)-1 and -2 (Shpak *et al*., 2004). To determine whether fertility differences between Col-0 and L*er rga gai* mutants relate to the *ER* locus, the Col-0 null allele *er-105* (Torii *et al*., 1996) was introgressed into *rga-28 gai-td1* (Col-0). The introduction of *er-105* resulted in an inflorescence phenotype resembling L*er* in all mutant combinations (Figs3g, S5), but *er-105 rga-28 gai-td1* remained infertile (Fig.3g). Flowers of *er-105* (Fig.3h,l), *er-105 rga-28* (Fig.3i,m) and *er-105 gai-td1* (Fig.3j,n) produced pollen and were self-fertilizing, but *er-105 rga-28 gai-td1* flowers were pollenless (Fig.3k,o). Fertility differences between Col-0 and L*er rga gai* mutants do not appear to be associated with *ER*.

### *rga-28 gai-td1* (Col-0) male sterility is caused by a post-meiotic defect in pollen development

**Figure 4:**
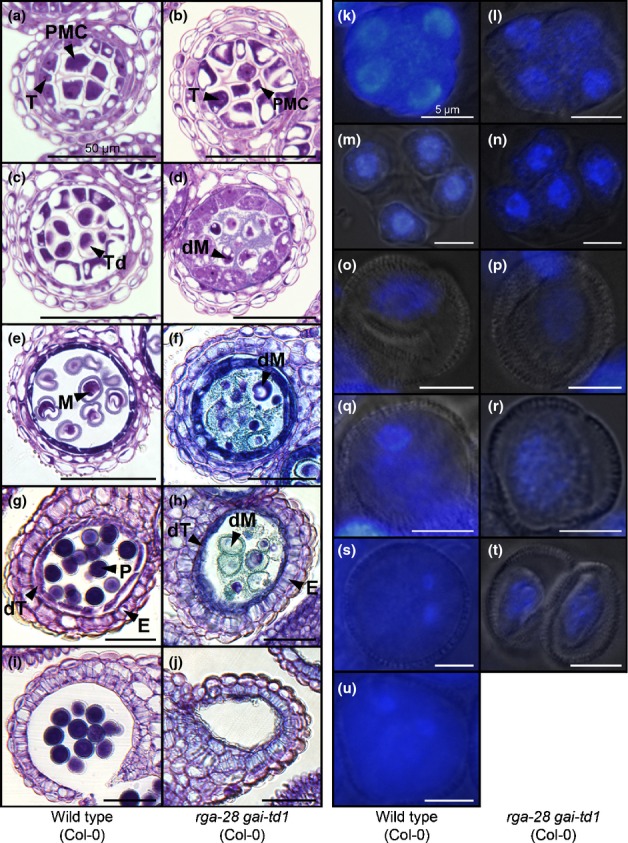
Microscopic analysis of *rga-28 gai-td1* (*Arabidopsis thaliana* Col-0) pollen development. (a–j) Sections through wild-type (a, c, e, g, i) and *rga-28 gai-td1* (Col-0) (b, d, f, h, j) anthers, encompassing developmental stages 5–6 (a, b), 7 (c, d), 8–9 (e, f), 11 (g, h) and 13 (i, j), as defined by Sanders *et al*. (1999). E, endothecium; dM, degenerating microspore; dT, degenerating tapetum; M, microspore; P, pollen; PMC, pollen mother cell; T, tapetum; Td, tetrad. (k–u) 4′,6-Diamidino-2-phenylindole (DAPI) fluorescence imaging of pollen nuclei in wild-type (k, m, o, q, s, u) and *rga-28 gai-td1* (Col-0) (l, n, p, r, t) during development, showing tetrad formation (k–n), free unicellular microspores (o, p), polarized microspores (q), bicellular pollen (s) and mature tricellular pollen (u). *rga-28 gai-td1* (Col-0) microspores do not polarize (p), and subsequently degenerate (r, t).

Anther and pollen development in *rga-28 gai-td1* (Col-0) were examined microscopically to identify the cause of pollen lethality. Pollen mother cells (PMCs) were visible in both wild-type and mutant anthers (Fig.4a,b). Successful tetrad formation and microspore release were observed in wild-type anthers (Fig.4c,m), whereas, by contrast, although some tetrads in *rga-28 gai-td1* anthers apparently progressed successfully to the free microspore stage (Fig.4n), abnormal meiotic products were frequently observed (Figs4d, S6). Post-meiotic *rga-28 gai-td1* pollen development was also abnormal: wild-type microspores progressed through pollen mitosis to maturity (Fig.4o,q,s,u), but microspore nuclear polarization before pollen mitosis was not observed in *rga-28 gai-td1* (Fig.4p), and microspores subsequently degenerated (Fig.4r,t).

At the free microspore stage of anther development (stages 8–9; Sanders *et al*., 1999), large amounts of particulate material accumulated in *rga-28 gai-td1* locules (Fig.4f,h), suggestive of defective pollen wall formation. *rga-28 gai-td1* tapetal cells were enlarged relative to their wild-type equivalents (Fig.4a–d), and the onset of tapetal breakdown appeared to be slightly delayed (Fig.4e,f). Both microspores and tapetum had degenerated in *rga-28 gai-td1* anthers by stage 11 (Fig.4h). The endothecium underwent apparently normal secondary thickening in *rga-28 gai-td1* anthers (Fig.4h,j). We also observed that synchronous development between locules within the same anther was lost in some *rga-28 gai-td1* stamens (Fig. S6), suggesting that DELLA activity might act to coordinate development between locules.

### Pollen lethality in *rga-28 gai-td1* is complemented by the reintroduction of RGA into either the tapetum or developing microspore

**Figure 5:**
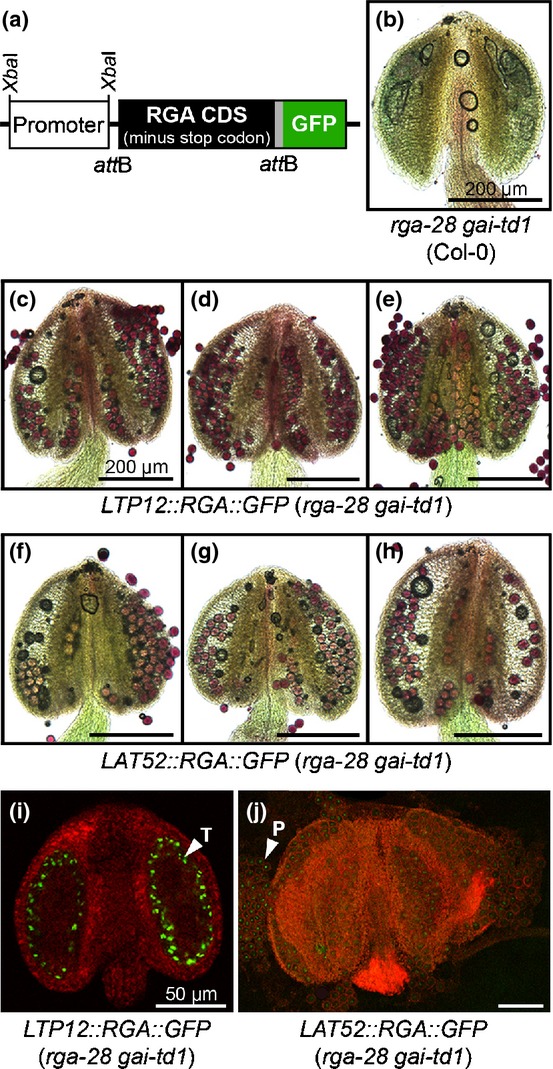
Reintroduction of REPRESSOR OF ga1-3 (RGA) rescues *Arabidopsis thaliana* pollen development in *rga-28 gai-td1*. (a) Schematic diagram of RGA-green fluorescent protein (GFP) transgenic fusion protein. (b) *rga-28 gai-td1* anther phenotype. (c–e) Anther phenotype of three independent *LTP12::RGA::GFP* (*rga-28 gai-td1*) transgenic lines. (f–h) Anther phenotype of three independent *LAT52::RGA::GFP* (*rga-28 gai-td1*) transgenic lines. Anthers were stained to determine pollen viability: dark red colouring indicates viable pollen. (i, j) Anther tissue localization of RGA-GFP under the *LTP12* (i) and *LAT52* (j) promoters. P, pollen; T, tapetum.

To confirm the requirement for DELLA activity for successful pollen development, and to identify the site of DELLA activity within the developing anthers, complementation of the *rga-28 gai-td1* phenotype was attempted by expressing GFP-tagged RGA (Fig.5a) in two separate anther tissues under the tapetally expressed Arabidopsis *LIPID TRANSFER PROTEIN 12* (*LTP12*) promoter (Ariizumi *et al*., 2002) or the tomato *LAT52* promoter (Twell *et al*., 1989), expressed in developing pollen (Twell *et al*., 1990; Eady *et al*., 1994; Roy *et al*., 2011). A total of 27 *LTP12::RGA::GFP* and 29 *LAT52::RGA::GFP* transgenic T_1_ plants were isolated in the *rga-28 gai-td1* background from three separate transformations with each construct. To our surprise, T_1_ plants expressing either construct in the *rga-28 gai-td1* homozygous mutant background exhibited self-fertility. Three independent homozygous lines carrying single T-DNA insertions were established for each construct and the expression of each transgene in inflorescence tissue was verified by reverse transcription-polymerase chain reaction (RT-PCR) (Fig. S7).

The fertility of T_3_ individuals was variable, but generally reduced compared with the wild-type early in flowering, with silique set recovering more robustly later (Fig. S8). T_1_ individuals generally set copious siliques and seed, and therefore reduced fertility exhibited by subsequent generations might be caused by silencing. In contrast with pollenless *rga-28 gai-td1* anthers (Fig.5b), viable pollen was observed in mature anthers of all lines (Fig.5c–h). Tissue-specific expression of *RGA-GFP* was tested by fluorescence microscopy. In *LTP12* lines, strong post-meiotic GFP fluorescence was detected exclusively in the tapetum (Fig.5i), with no visible signal in the developing pollen or other anther tissues. GFP intensity under *LAT52* expression was far weaker than in *LTP12*, even with stabilization of RGA-GFP using PAC treatment (see the Materials and Methods section). GFP fluorescence in *LAT52* transgenic lines was detected only in the nuclei of developing pollen (Fig.5j), supported by comparison against autofluorescence in wild-type anthers under the same confocal settings (Fig. S9). *LAT52::RGA::GFP* anthers were examined for tapetal GFP expression in earlier anther developmental stages, but no fluorescence above background levels was observed (Fig. S9). Segregating fluorescence in the pollen of hemizygous T_2_ individuals (Fig. S9) supports the assumption of exclusively post-meiotic expression under *LAT52*. These data suggest that independent expression of DELLA protein in either developing pollen or the surrounding tapetal cells is sufficient to rescue pollen development and fertility of *rga-28 gai-td1* (Col-0).

## Discussion

### Loss of the DELLA paralogues *RGA* and *GAI* affects Arabidopsis fertility more seriously in the Col-0 ecotype than in L*er*

The majority of past genetic analysis of Arabidopsis DELLA function has been conducted using loss-of-function mutants in the L*er* ecotype, the results of which suggested that DELLA activity is not strictly required to maintain fertility (Dill & Sun, 2001; Fuentes *et al*., 2012) and that DELLA proteins act simply to inhibit Arabidopsis floral and stamen development until alleviated by GA signalling (Dill & Sun, 2001; Cheng *et al*., 2004; Tyler *et al*., 2004). In this article, we describe a novel, male-sterile phenotype associated with loss of functional RGA and GAI in the Col-0 ecotype, in contrast with published *rga gai* mutants in L*er*, which retain some, albeit reduced, fertility (Dill & Sun, 2001).

The Col-0 phenotype corresponds closely to that in rice and barley, where loss of the sole DELLA orthologue results in sterility (Lanahan & Ho, 1988; Ikeda *et al*., 2001). The barley mutant, *slender1*, has been described as pollenless (Lanahan & Ho, 1988), which was also found to be the cause of sterility in the Col-0 mutant *rga-28 gai-td1*. This suggests a conserved requirement for DELLA activity in maintaining pollen development between dicots and monocots that was not previously appreciated based solely on the analysis of L*er* phenotypes. A closer inspection of two L*er rga gai* mutants identified a partial sterility phenotype, with individual anther locules devoid of pollen, suggesting that RGA and GAI regulate the same anther developmental processes in both Col-0 and L*er*, but their loss in L*er* has far less consequence for fertility. A similar phenomenon was observed under exogenous GA treatment, with the fertility of wild-type L*er* inflorescences remaining much higher than those of Col-0. The GA responses of Col-0 and L*er* also differed in other developmental pathways, most obviously in inflorescence architecture. Although some differential GA responses between Col-0 and L*er* might be explicable through starting differences in the levels of endogenous GA, their phenotypes remained significantly different under otherwise saturating GA concentrations (as indicated by the phenotypic similarity of GA-treated Col-0, *35S::GA20ox1* and *ga2ox*). Furthermore, altered GA content cannot explain the continued fertility of the L*er* DELLA *global* mutant (Fuentes *et al*., 2012), where all DELLA repression of downstream GA signalling has supposedly been lost.

Neither GA treatment nor genetic manipulation of GA biosynthesis replicated the phenotypic severity of *rga gai* mutants, probably because the DELLA protein is never completely depleted by GA signalling *in planta*, a hypothesis supported by past experimental studies (Tyler *et al*., 2004; Feng *et al*., 2008). GA signal transduction and GA biosynthesis are linked through homeostatic regulation (Zentella *et al*., 2007), with *DELLA* expression up-regulated by GA signalling in both rice and Arabidopsis (Itoh *et al*., 2002; Ariizumi *et al*., 2008). The hypothesis that reduced fertility under GA treatment can be explained by a purely mechanical barrier to pollination was not supported by the modelling of Col-0 floral organ growth. Instead, it was found that anther size is specifically reduced under GA treatment, suggesting that disrupted anther development could underlie the fertility effects of both GA overdose and loss of DELLA function. DELLA loss-of-function mutants, including the L*er global* mutant, also demonstrate a reduction (but not complete loss) specifically in female fertility (Dorcey *et al*., 2009; Fuentes *et al*., 2012). As such, the reductions in seed set observed outside of male-sterile lines in this study were probably caused both by impaired male and female reproductive processes.

### DELLA expression differs between Col-0 and L*er* stamen tissues

Arabidopsis carries five DELLA paralogues, the expression patterns of which are unknown. The differences in fertility between Col-0 and L*er rga gai* mutants might be caused by the expression of at least one of the remaining DELLA paralogues, *RGA-LIKE* (*RGL*)*1*,*RGL2* or *RGL3*, in L*er* anthers to maintain pollen development. The additional presence of RGL1 and RGL2 (but not RGL3) in developing L*er* pollen is supported by transcriptome data (Honys & Twell, 2004; Table S4), and loss of RGL2 has been shown to reduce pollen number in L*er* anthers and also to significantly rescue pollen production in the partially GA-insensitive *gai-1* (L*er*) background (Kay *et al*., 2013).

A comparison of past analyses highlights differences in the functional importance of DELLA paralogues between Col-0 and L*er* during floral development. In the GA-deficient *ga1-3* (L*er*) background, loss of functional RGA and GAI is not sufficient to overcome microspore developmental arrest (Dill & Sun, 2001; King *et al*., 2001; Cheng *et al*., 2004), requiring the combined loss of RGA, RGL1 and RGL2 (Cheng *et al*., 2004). By contrast, loss of RGA alone was sufficient to overcome arrested microspore development in *ga1-3* (Col-0) (Tyler *et al*., 2004), suggesting greater functional redundancy between DELLA paralogues during L*er* stamen development. Successful pollen production by the *rga-28 gid1* quadruple mutant, in contrast with pollenless *rga-28 gai-td1 gid1*, argues for a function for GAI in Col-0 pollen development redundant with RGA. Reduced floral organ growth in *rga-28 gai-td1 gid1* relative to *rga-28 gai-td1*, including the stamen filament, suggests that at least one other DELLA paralogue regulates Col-0 floral development. RGA and GAI were found to be the dominant DELLA paralogues regulating Col-0 vegetative development (Fig. S10), as also observed in L*er* (Dill & Sun, 2001; Cheng *et al*., 2004; Tyler *et al*., 2004).

### Differences apparently exist between the GA responses of Col-0 and L*er* beneath the level of DELLA activity

The L*er* DELLA *global* mutant, which carries mutations in all five paralogues, but nevertheless remains partially fertile (Fuentes *et al*., 2012), suggests that at least some differences in the *rga gai* pollen phenotype observed between Col-0 and L*er* are caused by genetic variation downstream of the DELLA proteins. Successful pollen production by the *global* mutant, albeit reduced, was confirmed by this study (Fig. S4). The theoretical possibility remains that the *global* mutant expresses some DELLA protein: the T-DNA insertion in the *rgl1-1* allele disrupts the promoter region, but not the coding sequence (Lee *et al*., 2002). As yet, no Col-0 equivalent to the *global* mutant is available for comparison.

Significant genomic variation has been reported between Col-0 and L*er*, including altered gene transcripts and gene expression patterns (Gan *et al*., 2011), some of which could potentially relate to GA signal transduction or downstream response pathways. ER protein itself indirectly regulates responses downstream of GA signalling, antagonizing SHORT INTERNODES (SHI), a zinc-finger transcription factor that negatively regulates GA responses (Fridborg *et al*., 2001). However, loss of functional ER did not rescue fertility or pollen development in the *rga-28 gai-td1* (Col-0) background.

### Loss of DELLA activity in Arabidopsis anthers disrupts post-meiotic pollen development

The defects in pollen production in both Col-0 and L*er rga gai* mutants suggest that DELLA activity in stamens does not merely inhibit development, but is also necessary for successful pollen production. Potentially, DELLA proteins might coordinate development between different anther tissues through inhibition at developmental ‘checkpoints’ until the necessary conditions to proceed are met. Pollen is heavily dependent on the surrounding tapetum for nutrition and the production of pollen wall components (Scott *et al*., 2004), and disruption of tapetal PCD results in pollen abortion (Kawanabe *et al*., 2006). Experiments in rice have demonstrated that GA signalling regulates both tapetal PCD and pollen wall synthesis (Aya *et al*., 2009). Cheng *et al*. (2004) identified malformed pollen in the *ga1-3 rga-t2 gai-t6 rgl1-1 rgl2-1* (L*er*) mutant, which they suggested was caused by the overproduction of pollen wall material. Coordination of anther development by DELLA is supported by our observation of desynchronized development between locules in *rga-28 gai-td1* anthers (Fig. S7), which might explain the locule specificity of male sterility in *rga gai* (L*er*) mutants. DELLA activity might also positively regulate pollen development directly: in floral tissues, 43% of DELLA transcriptional targets were up-regulated by the induction of DELLA expression (Cao *et al*., 2006).

*rga-28 gai-td1* anthers displayed defects in both microspore and tapetum developmental processes. Phenotypically normal tetrads and free microspores were observed, although other defective meiotic products suggest perturbation to meiosis in some instances. Aberrant pollen development in *rga-28 gai-td1* first becomes visible at the polarized microspore stage. Although the precise cause of pollen lethality in *rga-28 gai-td1* could not be determined, the accumulation of free material in mutant locules is suggestive of defective pollen wall formation through aberrant tapetum function. However, whether this phenomenon is causative remains unclear, and potential intrinsic defects in microspore development (or a combination of both microspore and tapetal defects) should not be excluded. In rice pollen, *SLENDER RICE1* (*SLR1*) is up-regulated specifically during meiosis and in tetrads (Hirano *et al*., 2008; Tang *et al*., 2010), suggesting that GA signalling is tightly regulated during meiosis. Similar resolution is not yet available for Arabidopsis, but *AtDELLA* expression has been identified in both unicellular and bicellular L*er* pollen (Honys & Twell, 2004; Table S4). Arabidopsis pollen development was rescued by the reintroduction of RGA under two separate promoters, *LTP12* and *LAT52*, both of which first express in post-meiotic tissues at or before the point at which the mutant pollen phenotype manifests, during the unicellular microspore stage (*LTP12*, Ariizumi *et al*., 2002) and in individual microspores within tetrads (*LAT52*, Francis *et al*., 2007). These indicate that, at the least, microspore development can be rescued by the reintroduction of DELLA signalling beyond the completion of meiosis. Other anther wall processes, such as endothecium secondary thickening and stomium development, occurred in a timely fashion in the *rga-28 gai-td1* locule, suggesting that anther wall maturation is not exclusively dependent on RGA and GAI, but the possibility cannot be excluded that normal anther wall development was maintained by other DELLA paralogues.

### Expression of RGA in either the tapetum or developing pollen rescued *rga-28 gai-td1* fertility

Suppression of pollen lethality in *rga-28 gai-td1* under either promoter was surprising, as each has been shown previously to express exclusively in separate tissues, *LTP12* in the tapetum (Ariizumi *et al*., 2002) and *LAT52* in developing pollen (Twell *et al*., 1990; Eady *et al*., 1994; Francis *et al*., 2007; Roy *et al*., 2011). Although the expression domains of native DELLAs in Arabidopsis anther tissues are unresolved, in rice, *SLR1* is expressed simultaneously in both the tapetum and pollen (Hirano *et al*., 2008; Tang *et al*., 2010). No fluorescence or phenotypic evidence (e.g. dwarfing; Fig. S8) was found to suggest that RGA was expressed outside of the previously published expression patterns for *LTP12* or *LAT52*. Nevertheless, the possibility cannot be entirely discounted that the observed complementation of defective pollen development under either promoter is an artefact caused by leaky basal expression in other anther tissues, and that DELLA activity is only required in the tapetum or pollen. One foreseeable mechanism for this could be inheritance by otherwise mutant microspores of DELLA protein from pre-meiotic anther cell lineages. A similar phenomenon has been hypothesized to explain the successful creation of theoretically lethal GA-insensitive *gid1* mutants (Griffiths *et al*., 2006). That said, segregation of *LAT52*-driven fluorescence was observed in pollen produced by hemizygous T_2_ individuals (Fig. S9), suggesting that *LAT52*-expressed RGA is not present in PMCs.

If the independence of *LTP12*- and *LAT52*-driven expression is accepted, the rescue of pollen development by RGA in either the tapetum or pollen suggests that communication occurs between these two cell types downstream of DELLA activity. Such communication has been demonstrated during cell fate specification, where the PMC-secreted ligand TAPETUM DETERMINANT 1 (TPD1) interacts with the tapetum receptor EXTRA SPOROGENOUS CELLS/EXCESS MICROSPOROCYTES (EXS/EMS) (Yang *et al*., 2003, 2005; Jia *et al*., 2008) and, later in development, microspores may communicate with surrounding somatic cells via the extracellular protein MTR1 (Tan *et al*., 2012). The *GAI* transcript itself has been reported as a mobile signal (Ruiz-Medrano *et al*., 1999; Haywood *et al*., 2005).

The outcome of these comparative analyses is that it can no longer be assumed that experimental results from one ecotype are necessarily representative of all. Ecotypic differences also represent an additional and useful tool for the elucidation of genetic pathways, such as those underlying GA regulation of Arabidopsis pollen development.

## Supporting Information

Fig S1 Loss of *REPRESSOR OF ga1-3* (*RGA*) and *GIBBERELLIN INSENSITIVE* (*GAI*) causes infertility in the Col-0 background. **Fig. S2** Effect of gibberellin (GA) treatment on silique set and floral organ growth. **Fig. S3** Comparison of *gid1a-1 gid1b-1 gid1c-1* and *gid1a-1 gid1b-1 gid1c-2* phenotypes. **Fig. S4** The L*er* DELLA *global* mutant retains pollen viability. **Fig. S5** Loss of *ERECTA* (*ER*) in the *rga-28 gai-td1* (Col-0) background phenocopies the L*er* growth habit. **Fig. S6** Additional anther and pollen phenotypes of *rga-28 gai-td1*. **Fig. S7** Reverse transcription-polymerase chain reaction (RT-PCR) analysis of REPRESSOR OF ga1-3-green fluorescent protein (RGA-GFP) expression in *LTP12::RGA::GFP* and *LAT52::RGA::GFP* transgenic lines. **Fig. S8** Vegetative and reproductive phenotypes of *LTP12::RGA::GFP* and *LAT52::RGA::GFP* transgenic lines. **Fig. S9** Fluorescence analysis of *LTP12::RGA::GFP* and *LAT52::RGA::GFP* expression. **Fig. S10** Genetic analysis of *rga gai gid1* vegetative phenotypes. **Table S1** PCR primer sequences **Table S2** Col-0 and L*er* respond differently to chemical and genetic gibberellin (GA) overdose **Table S3** Modelling the effect of gibberellin (GA) treatment on floral organ growth in wild-type Col-0 **Table S4** Expression of gibberellin (GA) biosynthetic and signalling genes during wild-type L*er* pollen development **Methods S1** Non-linear modelling of floral organ growth.

## References

[b1] Alexander MP (1969). Differential staining of aborted and non-aborted pollen. Stain Technology.

[b2] Alonso JM, Stepanova AN, Leisse TJ, Kim CJ, Chen H, Shinn P, Stevenson DK, Zimmerman J, Barajas P, Cheuk R (2003). Genome-wide insertional mutagenesis of *Arabidopsis thaliana*. Science.

[b3] Ariizumi T, Amagai M, Shibata D, Hatakeyama K, Watanabe M, Toriyama K (2002). Comparative study of promoter activity of three anther-specific genes encoding lipid transfer protein, xyloglucan endotransglucosylase/hydrolase and polygalacturonase in transgenic *Arabidopsis thaliana*. Plant Cell Reports.

[b4] Ariizumi T, Murase K, Sun T-P, Steber CM (2008). Proteolysis-independent downregulation of DELLA repression in *Arabidopsis* by the gibberellin receptor GIBBERELLIN INSENSITIVE DWARF1. Plant Cell.

[b5] Aya K, Ueguchi-Tanaka M, Kondo M, Hamada K, Yano K, Nishimura M, Matsuoka M (2009). Gibberellin modulates anther development in rice via the transcriptional regulation of GAMYB. Plant Cell.

[b6] Cao DN, Cheng H, Wu W, Soo HM, Peng JR (2006). Gibberellin mobilizes distinct DELLA-dependent transcriptomes to regulate seed germination and floral development in Arabidopsis. Plant Physiology.

[b8] Cheng H, Qin LJ, Lee SC, Fu XD, Richards DE, Cao DN, Luo D, Harberd NP, Peng JR (2004). Gibberellin regulates *Arabidopsis* floral development via suppression of DELLA protein function. Development.

[b9] Coles JP, Phillips AL, Croker SJ, García-Lepe R, Lewis MJ, Hedden P (1999). Modification of gibberellin production and plant development in *Arabidopsis* by sense and antisense expression of gibberellin 20-oxidase genes. Plant Journal.

[b10] Dill A, Sun T-P (2001). Synergistic derepression of gibberellin signaling by removing RGA and GAI function in *Arabidopsis thaliana*. Genetics.

[b11] Dorcey E, Urbez C, Blázquez M, Carbonell J, Perez-Amador M (2009). Fertilization-dependent auxin response in ovules triggers fruit development through the modulation of gibberellin metabolism in Arabidopsis. Plant Journal.

[b12] Eady C, Lindsey K, Twell D (1994). Differential activation and conserved vegetative cell-specific activity of a late pollen promoter in species with bicellular and tricellular pollen. Plant Journal.

[b13] Feng SH, Martinez C, Gusmaroli G, Wang Y, Zhou JL, Wang F, Chen LY, Yu L, Iglesias-Pedraz JM, Kircher S (2008). Coordinated regulation of *Arabidopsis thaliana* development by light and gibberellins. Nature.

[b14] Francis KE, Lam SY, Harrison BD, Bey AL, Berchowitz LE, Copenhaver GP (2007). Pollen tetrad-based visual assay for meiotic recombination in *Arabidopsis*. Proceedings of the National Academy of Sciences, USA.

[b15] Fridborg I, Kuusk S, Robertson M, Sundberg E (2001). The Arabidopsis protein SHI represses gibberellin responses in Arabidopsis and barley. Plant Physiology.

[b16] Fuentes S, Ljung K, Sorefan K, Alvey E, Harberd NP, Østergaard L (2012). Fruit growth in *Arabidopsis* occurs via DELLA-dependent and DELLA-independent gibberellin responses. Plant Cell.

[b17] Gan XC, Stegle O, Behr J, Steffen JG, Drewe P, Hildebrand KL, Lyngsoe R, Schultheiss SJ, Osborne EJ, Sreedharan VT (2011). Multiple reference genomes and transcriptomes for *Arabidopsis thaliana*. Nature.

[b18] Gomez KA, Gomez AA (1984). Statistical procedures for agricultural research.

[b19] Goto N, Pharis RP (1999). Role of gibberellins in the development of floral organs of the gibberellin-deficient mutant, *ga1-1*, of *Arabidopsis thaliana*. Canadian Journal of Botany.

[b20] Griffiths J, Murase K, Rieu I, Zentella R, Zhang ZL, Powers SJ, Gong F, Phillips AL, Hedden P, Sun T-P (2006). Genetic characterisation and functional analysis of the GID1 gibberellin receptors in *Arabidopsis*. Plant Cell.

[b21] Harberd NP, Belfield E, Yasumura Y (2009). The angiosperm gibberellin-GID1-DELLA growth regulatory mechanism: how an “inhibitor of an inhibitor” enables flexible response to fluctuating environments. Plant Cell.

[b22] Haywood V, Yu TS, Huang NC, Lucas WJ (2005). Phloem long-distance trafficking of *GIBBERELLIC ACID-INSENSITIVE* RNA regulates leaf development. Plant Journal.

[b23] Hirano K, Aya K, Hobo T, Sakakibara H, Kojima M, Shim RA, Hasegawa Y, Ueguchi-Tanaka M, Matsuoka M (2008). Comprehensive transcriptome analysis of phytohormone biosynthesis and signaling genes in microspore/pollen and tapetum of rice. Plant and Cell Physiology.

[b24] Honys D, Twell D (2004). Transcriptome analysis of haploid male gametophyte development in *Arabidopsis*. Genome Biology.

[b25] Hou XL, Hu WW, Shen L, Lee LYC, Tao Z, Han JH, Yu H (2008). Global identification of DELLA target genes during Arabidopsis flower development. Plant Physiology.

[b26] Hu JH, Mitchum MG, Barnaby N, Ayele BT, Ogawa M, Nam E, Lai WC, Hanada A, Alonso JM, Ecker JR (2008). Potential sites of bioactive gibberellin production during reproductive growth in *Arabidopsis*. Plant Cell.

[b27] Ikeda A, Ueguchi-Tanaka M, Sonoda Y, Kitano H, Koshioka M, Futsuhara Y, Matsuoka M, Yamaguchi J (2001). *slender* rice, a constitutive gibberellin response mutant, is caused by a null mutation of the *SLR1* gene, an ortholog of the height-regulating gene *GAI**RGA**RHT**D8*. Plant Cell.

[b28] Itoh H, Ueguchi-Tanaka M, Sato Y, Ashikari M, Matsuoka M (2002). The gibberellin signaling pathway is regulated by the appearance and disappearance of SLENDER RICE1 in nuclei. Plant Cell.

[b29] Jacobsen SE, Olszewski NE (1991). Characterization of the arrest in anther development associated with gibberellin deficiency of the *gib-1* mutant of tomato. Plant Physiology.

[b30] Jacobsen SE, Olszewski NE (1993). Mutations at the *SPINDLY* locus of Arabidopsis alter gibberellin signal transduction. Plant Cell.

[b31] Jia GX, Liu XD, Owen HA, Zhao DZ (2008). Signaling of cell fate determination by the TPD1 small protein and EMS1 receptor kinase. Proceedings of the National Academy of Sciences, USA.

[b32] Kawanabe T, Ariizumi T, Kawai-Yamada M, Uchimiya H, Toriyama K (2006). Abolition of the tapetum suicide program ruins microsporogenesis. Plant and Cell Physiology.

[b33] Kay P, Groszmann M, Ross JJ, Parish RW, Swain SM (2013). Modifications of a conserved regulatory network involving INDEHISCENT controls multiple aspects of reproductive tissue developments in Arabidopsis. New Phytologist.

[b34] King KE, Moritz T, Harberd NP (2001). Gibberellins are not required for normal stem growth in *Arabidopsis thaliana* in the absence of GAI and RGA. Genetics.

[b35] Lanahan MB, Ho THD (1988). Slender barley: a constitutive gibberellin-response mutant. Planta.

[b36] Lee SC, Cheng H, King KE, Wang WF, He Y, Hussain A, Lo J, Harberd NP, Peng JR (2002). Gibberellin regulates Arabidopsis seed germination via *RGL2*, a *GAI/RGA*-like gene whose expression is up-regulated following imbibition. Genes & Development.

[b37] de Lucas M, Daviere JM, Rodríguez-Falcón M, Pontin M, Iglesias-Pedraz JM, Lorrain S, Fankhauser C, Blázquez MA, Titarenko E, Prat S (2008). A molecular framework for light and gibberellin control of cell elongation. Nature.

[b38] McCullagh P, Nelder JA (1989). Generalised linear models.

[b39] Millar AA, Gubler F (2005). The Arabidopsis *GAMYB**like* genes, *MYB33* and *MYB65*, are microRNA-regulated genes that redundantly facilitate anther development. Plant Cell.

[b40] Nakagawa T, Suzuki T, Murata S, Nakamura S, Hino T, Maeo K, Tabata R, Kawai T, Tanaka K, Niwa Y (2007). Improved gateway binary vectors: high-performance vectors for creation of fusion constructs in transgenic analysis of plants. Bioscience Biotechnology and Biochemistry.

[b41] Nester JE, Zeevaart JAD (1988). Flower development in normal tomato and a gibberellin-deficient (*ga-2*) mutant. American Journal of Botany.

[b42] Peng JR, Carol P, Richards DE, King KE, Cowling RJ, Murphy GP, Harberd NP (1997). The *Arabidopsis GAI* gene defines a signaling pathway that negatively regulates gibberellin responses. Genes & Development.

[b43] Pharis RP, King RW (1985). Gibberellins and reproductive development in seed plants. Annual Review of Plant Physiology.

[b44] Plackett ARG, Thomas SG, Wilson ZA, Hedden P (2011). Gibberellin control of stamen development: a fertile field. Trends in Plant Science.

[b45] Pysh LD, Wysocka-Diller JW, Camilleri C, Bouchez D, Benfey PN (1999). The GRAS gene family in Arabidopsis: sequence characterisation and basic expression analysis of the *SCARECROW-LIKE* genes. Plant Journal.

[b46] Ragni L, Nieminen K, Pacheco-Villalobos D, Sibout R, Schwechheimer C, Hardtke CS (2011). Mobile gibberellin directly stimulates *Arabidopsis* hypocotyl xylem expansion. Plant Cell.

[b47] Rieu I, Eriksson S, Powers SJ, Gong F, Griffiths J, Woolley L, Benlloch R, Nilsson O, Thomas SG, Hedden P (2008a). Genetic analysis reveals that C_19_-GA 2-oxidation is a major gibberellin inactivation pathway in *Arabidopsis*. Plant Cell.

[b48] Rieu I, Ruiz-Rivero O, Fernandez-Garcia N, Griffiths J, Powers SJ, Gong F, Linhartova T, Eriksson S, Nilsson O, Thomas SG (2008b). The gibberellin biosynthetic genes *AtGA20ox1* and *AtGA20ox2* act, partially redundantly, to promote growth and development throughout the Arabidopsis life cycle. Plant Journal.

[b49] Robinson SJ, Tang LH, Mooney BAG, McKay SJ, Clarke WE, Links MG, Karcz S, Regan S, Wu YY, Gruber MY (2009). An archived activation tagged population of *Arabidopsis thaliana* to facilitate forward genetics approaches. BMC Plant Biology.

[b50] Roy B, Copenhaver GP, von Arnim AG (2011). Fluorescence-tagged transgenic lines reveal genetic defects in pollen growth- application to the Eif3 complex. PLoS ONE.

[b51] Ruiz-Medrano R, Xoconostle-Cázares B, Lucas WJ (1999). Phloem long-distance transport of *CmNACP* mRNA: implications for supracellular regulation in plants. Development.

[b52] Sanders PM, Bui AQ, Weterings K, McIntire KN, Hsu YC, Lee PY, Truong MT, Beals TP, Goldberg RB (1999). Anther developmental defects in *Arabidopsis thaliana* male sterile mutants. Sexual Plant Reproduction.

[b53] Scott RJ, Spielman M, Dickinson HG (2004). Stamen structure and function. Plant Cell.

[b54] Sessions A, Burke E, Presting G, Aux G, McElver J, Patton D, Dietrich B, Ho P, Bacwaden J, Ko C (2002). A high-throughput Arabidopsis reverse genetics system. Plant Cell.

[b55] Shpak ED, Berthiaume CT, Hill EJ, Torii KU (2004). Synergistic interaction of three ERECTA-family receptor-like kinases controls *Arabidopsis* organ growth and flower development be promoting cell proliferation. Development.

[b56] Silverstone AL, Mak PYA, Martínez EC, Sun T-P (1997). The new *RGA* locus encodes a negative regulator of gibberellin response in *Arabidopsis thaliana*. Genetics.

[b57] Sun T-P (2010). Gibberellin-GID1-DELLA: a pivotal regulatory module for plant growth and development. Plant Physiology.

[b58] Tan H, Liang W, Hu J, Zhang D (2012). *MTR1* encodes a secretory fasciclin glycoprotein required for male reproductive development in rice. Developmental Cell.

[b59] Tang X, Zhang ZY, Zhang WJ, Zhao XM, Li X, Zhang D, Liu QQ, Tang WH (2010). Global gene profiling of laser-captured pollen mother cells indicates molecular pathways and gene subfamilies involved in rice meiosis. Plant Physiology.

[b60] Tarnowski BI, Spinale FG, Nicholson JH (1991). DAPI as a useful stain for nuclear quantitation. Biotechnic and Histochemistry.

[b61] Torii KU, Mitsukawa N, Oosumi T, Matsuura Y, Yokoyama R, Whittier RF, Komeda Y (1996). The Arabidopsis *ERECTA* gene encodes a putative receptor protein kinase with extracellular leucine-rich repeats. Plant Cell.

[b62] Twell D, Wing R, Yamaguchi J, McCormick S (1989). Isolation and expression of an anther-specific gene from tomato. Molecular Genetics and Genomics.

[b63] Twell D, Yamaguchi J, McCormick S (1990). Pollen-specific gene expression in transgenic plants: coordinate regulation of two different tomato gene promoters during microsporogenesis. Development.

[b64] Tyler L, Thomas SG, Hu JH, Dill A, Alonso JM, Ecker JR, Sun T-P (2004). DELLA proteins and gibberellin-regulated seed germination and floral development in Arabidopsis. Plant Physiology.

[b65] Ueguchi-Tanaka M, Matsuoka M (2010). The perception of gibberellins: clues from receptor structure. Current Opinion in Plant Biology.

[b66] Vizcay-Barrena G, Wilson ZA (2006). Altered tapetal PCD and pollen wall development in the *Arabidopsis ms1* mutant. Journal of Experimental Botany.

[b67] Willige BC, Ghosh S, Nill C, Zourelidou M, Dohmann EMN, Maier A, Schwechheimer C (2007). The DELLA domain of GA INSENSTIVE mediates the interaction with the GA INSENSITIVE DWARF1A gibberellin receptor of *Arabidopsis*. Plant Cell.

[b68] Yang SL, Jiang LX, Puah CS, Xie LF, Zhang XQ, Chen LQ, Yang WC, Ye D (2005). Overexpression of *TAPETUM DETERMINANT1* alters the cell fates in the Arabidopsis carpel and tapetum via genetic interaction with *EXCESS MICROSPOROCYTES1/EXTRA SPOROGENOUS CELLS*. Plant Physiology.

[b69] Yang SL, Xie LF, Mao HZ, Puah CS, Yang WC, Jiang LX, Sundaresan V, Ye D (2003). *TAPETUM DETERMINANT1* is required for cell specialization in the Arabidopsis anther. Plant Cell.

[b70] Zentella R, Zhang ZL, Park M, Thomas SG, Endo A, Murase K, Fleet CM, Jikumaru Y, Nambara E, Kamiya Y (2007). Global analysis of DELLA direct targets in early gibberellin signalling in *Arabidopsis*. Plant Cell.

