# Supplementary material for: DELLA activity is required for successful pollen development in the Columbia ecotype of Arabidopsis
Source: New Phytol. 2013 Nov 1;201(3):825–36. doi: 10.1111/nph.12571 (PMC4291109; doi:10.1111/nph.12571)
Supplement: Fig S1 — Loss of REPRESSOR OF ga1-3 (RGA) and GIBBERELLIN INSENSITIVE (GAI) causes infertility in the Col-0 background. Fig. S2 Effect of gibberellin (GA) treatment on silique set and floral organ growth. Fig. S3 Comparison of gid1a-1 gid1b-1 gid1c-1 and gid1a-1 gid1b-1 gid1c-2 phenotypes. Fig. S4 The Ler DELLA global mutant retains pollen viability. Fig. S5 Loss of ERECTA (ER) in the rga-28 gai-td1 (Col-0) background phenocopies the Ler growth habit. Fig. S6 Additional anther and pollen phenotypes of rga-28 gai-td1. Fig. S7 Reverse transcription-polymerase chain reaction (RT-PCR) analysis of REPRESSOR OF ga1-3-green fluorescent protein (RGA-GFP) expression in LTP12::RGA::GFP and LAT52::RGA::GFP transgenic lines. Fig. S8 Vegetative and reproductive phenotypes of LTP12::RGA::GFP and LAT52::RGA::GFP transgenic lines. Fig. S9 Fluorescence analysis of LTP12::RGA::GFP and LAT52::RGA::GFP expression. Fig. S10 Genetic analysis of rga gai gid1 vegetative phenotypes. Table S1 PCR primer sequences Table S2 Col-0 and Ler respond differently to chemical and genetic gibberellin (GA) overdose Table S3 Modelling the effect of gibberellin (GA) treatment on floral organ growth in wild-type Col-0 Table S4 Expression of gibberellin (GA) biosynthetic and signalling genes during wild-type Ler pollen development Methods S1 Non-linear modelling of floral organ growth. [file nph0201-0825-sd1.doc]

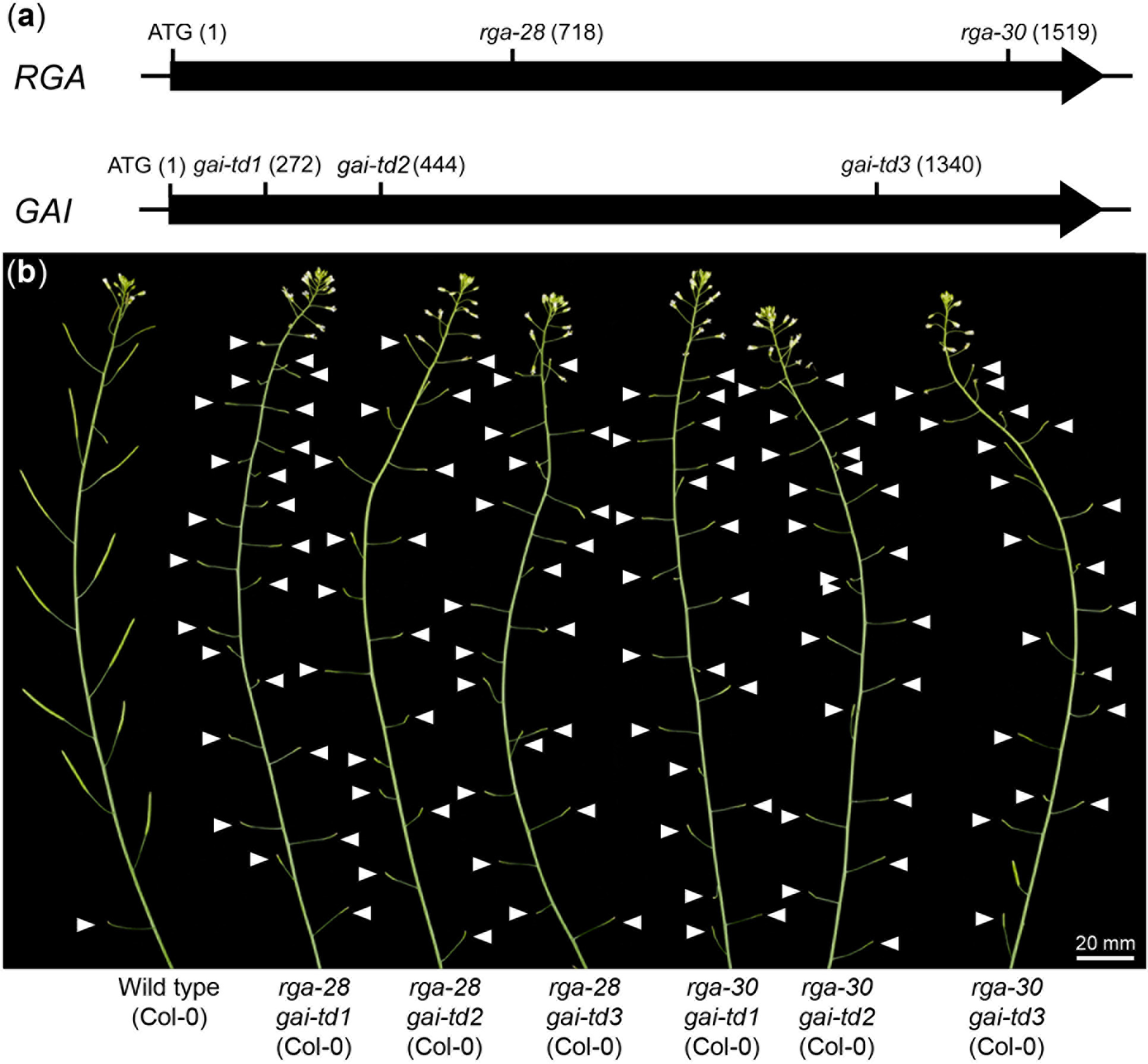


**Supporting Information Figs S1–S10, Tables S1–S4, Methods S1**

**Fig. S1.** Loss of *RGA* and *GAI* causes infertility in the Col-0 background.

(a) Gene models of *RGA* and *GAI*, with the positions of individual T-DNA insertions indicated. Positions given are with reference to the ATG.

(b) Primary inflorescence phenotypes of *rga gai* double mutant combinations compared against wild-type Col-0 at 32 days old. White arrows indicate infertile silique positions.


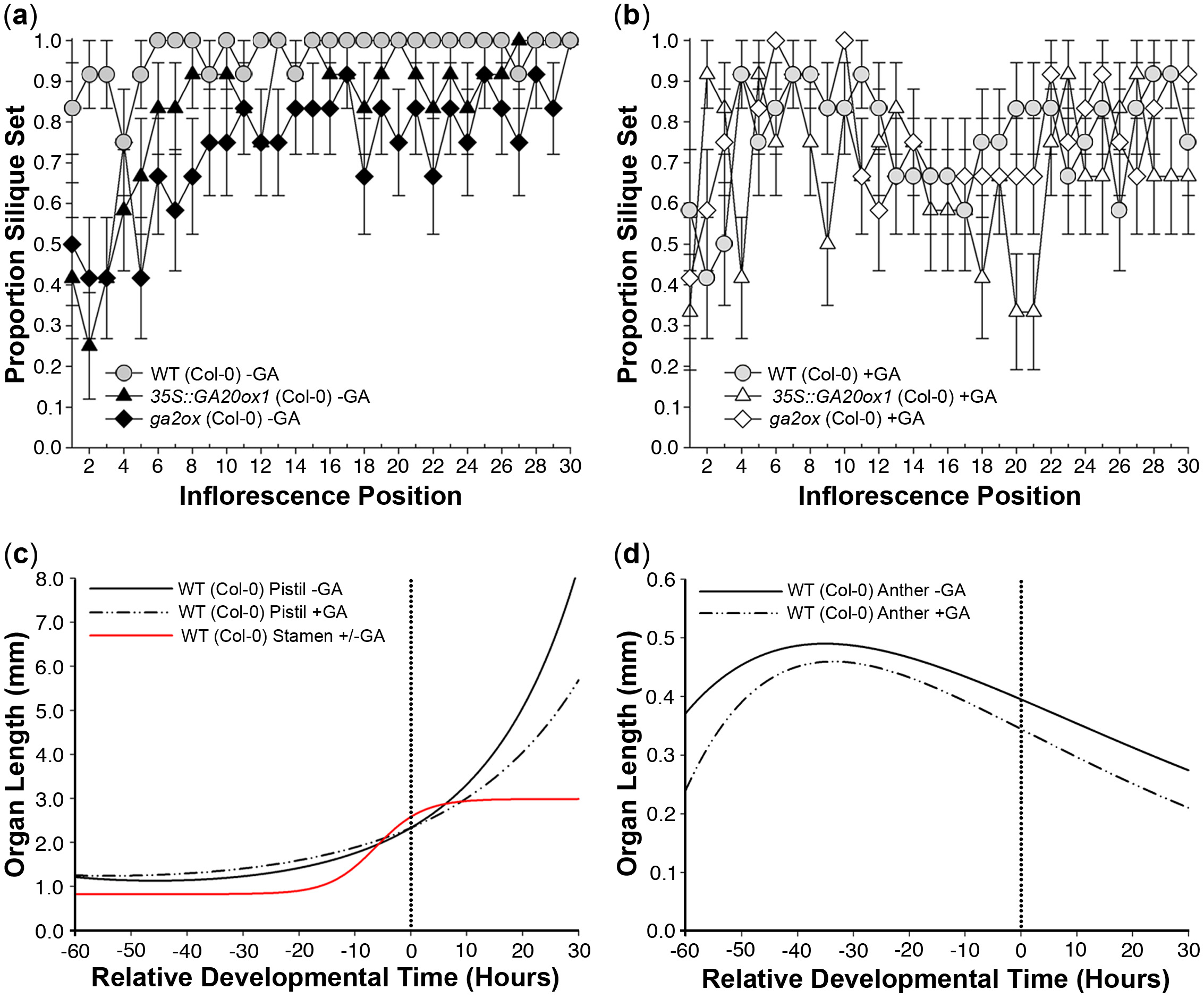


**Fig. S2** Effect of GA treatment on silique-set and floral organ growth.

(a),(b) Mean proportional frequency of silique-set across the primary inflorescence (*n* = 12, ±SE) under control (a) and GA-treated conditions (b), comparing wild-type Col-0 with *35S::GA20ox1* (Col-0) and the *ga2ox* (Col-0) quintuple loss-of-function mutant (see Materials & Methods). Presence or absence of a fertile silique was scored at each primary inflorescence position throughout flowering. Mean proportions of silique-set across flowering were measured for *35S::GA20ox1* as 0.84±0.04 (-GA) and 0.68±0.03 (+GA), and for *ga2ox* 0.74±0.03 (-GA) and 0.77±0.03 (+GA), respectively.

(c),(d) Best fit growth models of reproductive organs (c) and anthers (d) in wild-type Col-0 flowers during flower opening, under control growth conditions and GA treatment (model parameters supplied in Table S2, see Method S1). Dotted line indicates flower opening, developmental time represented as hours before or after flower opening.


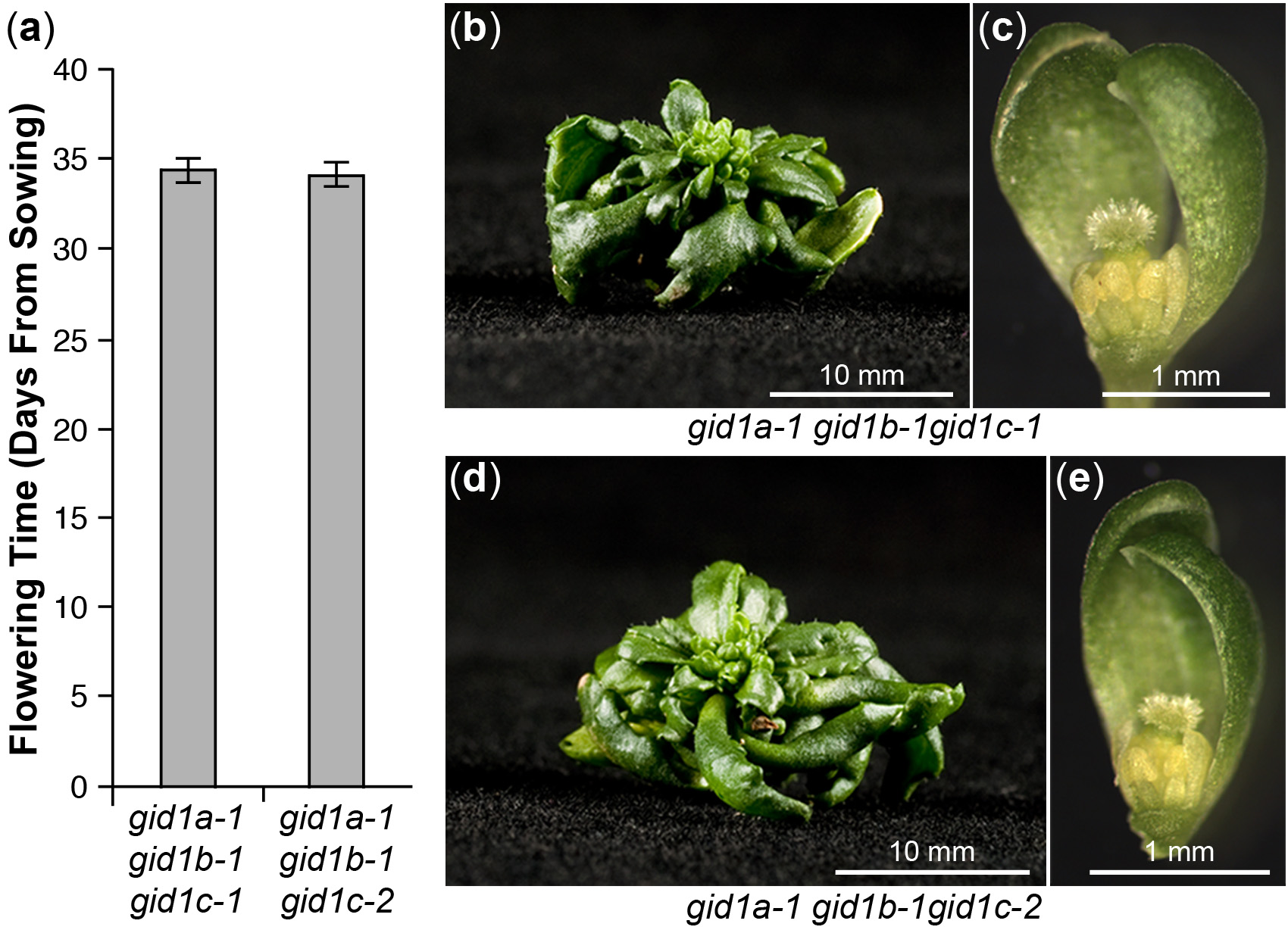


**Fig. S3** Comparison of *gid1a-1 gid1b-1 gid1c-1* and *gid1a-1 gid1b-1 gid1c-2* phenotypes.

(a) Mean flowering time (±SE) of the *gid1a-1 gid1b-1 gid1c-1* (*n* = 10) and *gid1a-1 gid1b-1 gid1c-2* (*n* = 11) triple mutants under LD growth conditions, Flowering time was not significantly different (p > 0.05, t-test).

(b)-(e) Comparison of whole rosette and floral phenotypes of *gid1a-1 gid1b-1 gid1c-1* ((b),(c)) and *gid1a-1 gid1b-1 gid1c-2* ((d),(e)).


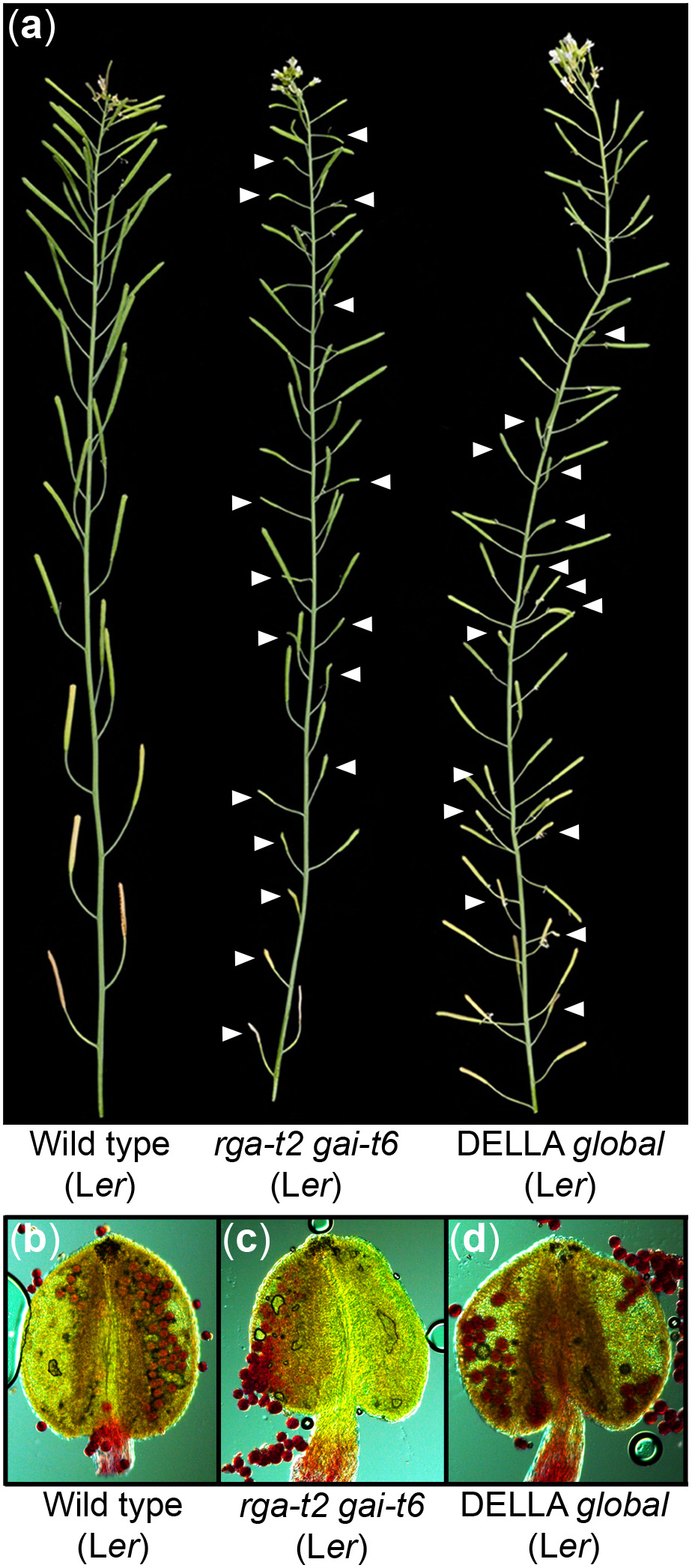


**Fig. S4.** The L*er* DELLA *global* mutant retains pollen viability.

(a) Fertility phenotypes of *rga-t2 gai-t6* and DELLA *global* mutant primary inflorescences compared against wild type L*er*. White arrows indicate infertile silique positions.

(b)-(d) Pollen viability of wild-type L*er* (b), *rga-t2 gai-t6* (c) and *global* (d): dark red colouring indicates viable pollen.


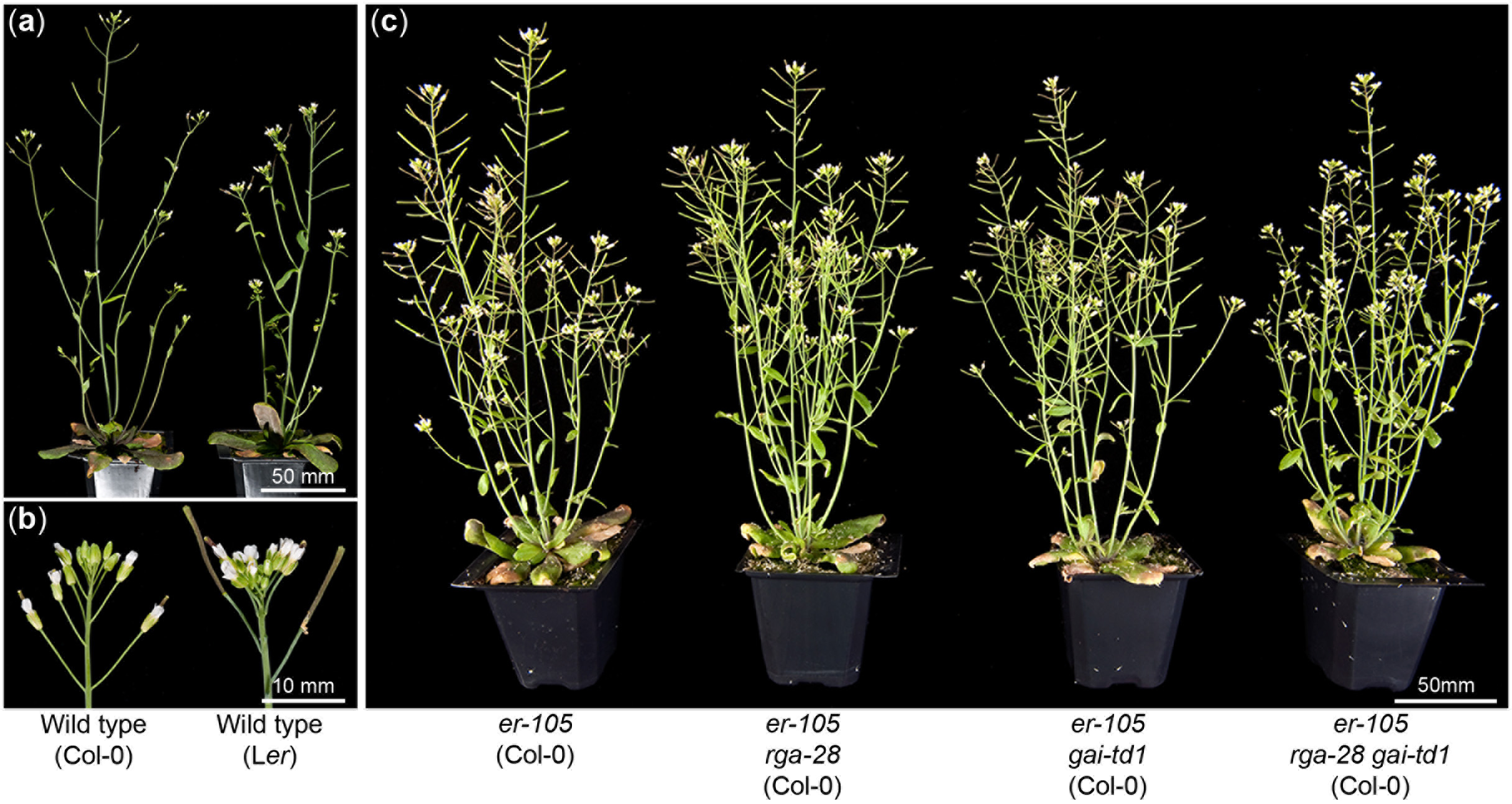


**Fig. S5.** Loss of *ER* in the *rga-28 gai-td1* (Col-0) background phenocopies the L*er* growth habit.

(a),(b) Comparison between wild type Col-0 and L*er* inflorescence architecture (a) and floral cluster architecture (b).

(c) Inflorescence architecture of *er-105 rga-28 gai-td1* (Col-0) combinatorial mutants.

Plants shown in (a)-(b) and (c) are 27 and 36 days old, respectively.


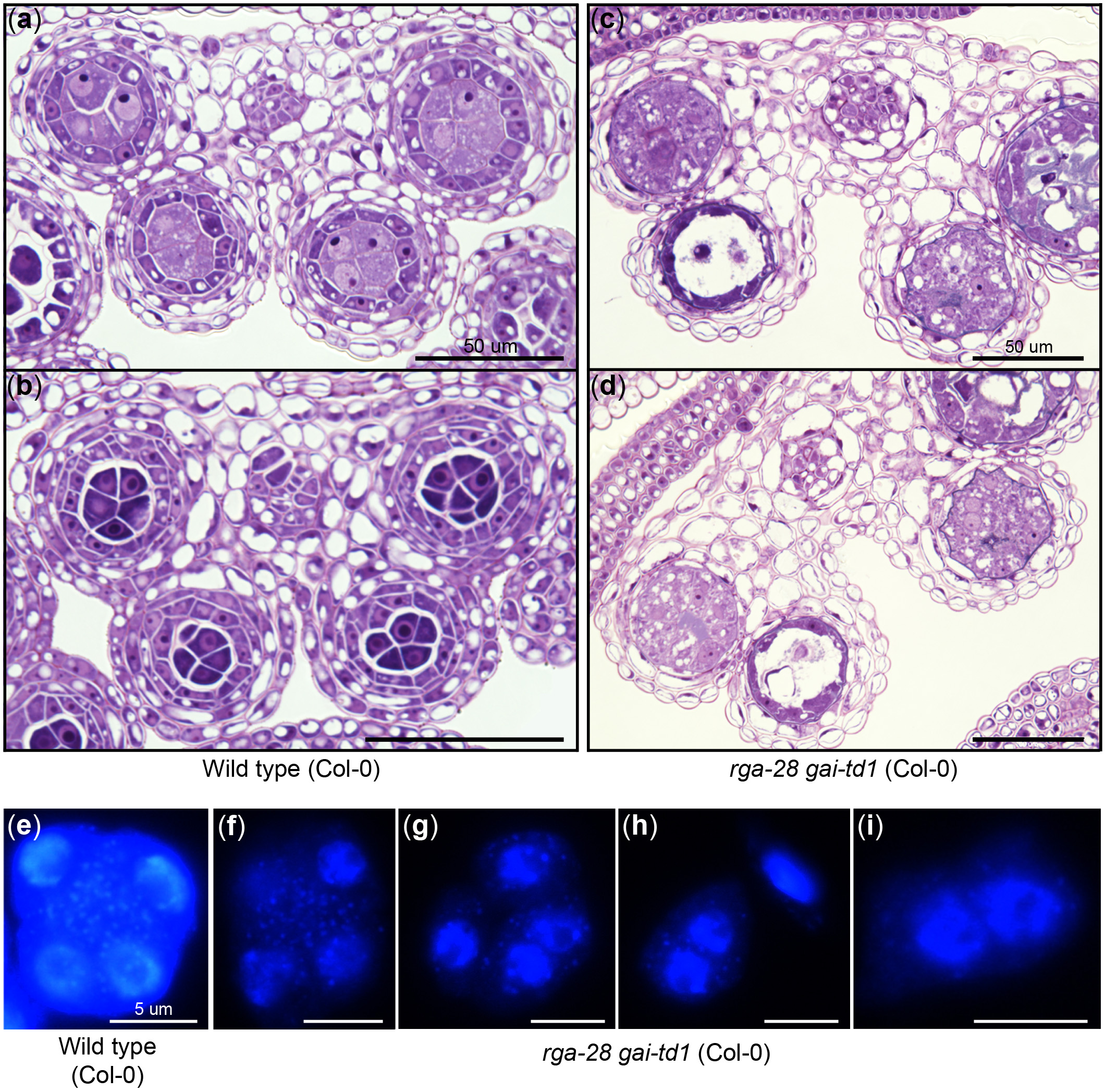


**Fig. S6.** Additional anther and pollen phenotypes of *rga-28 gai-td1*.

(a),(b) Wild-type (Col-0) anther sections, stage 5-6. All locules are at similar developmental stages.

(c),(d) *rga-28 gai-td1* (Col-0) anther sections. Whilst most locules are at anther stage 5-6, in each anther one locule has already progressed through to post-meiotic pollen development.

(e)-(i) DAPI fluorescence of wild-type Col-0 (e) and *rga-28 gai-td1* (Col-0) tetrads ((f)-(i)). Four meiotic products were observed in some mutant tetrads (f), whilst others exhibited abnormal product division resulting in apparently binucleate products ((g)-(i)).


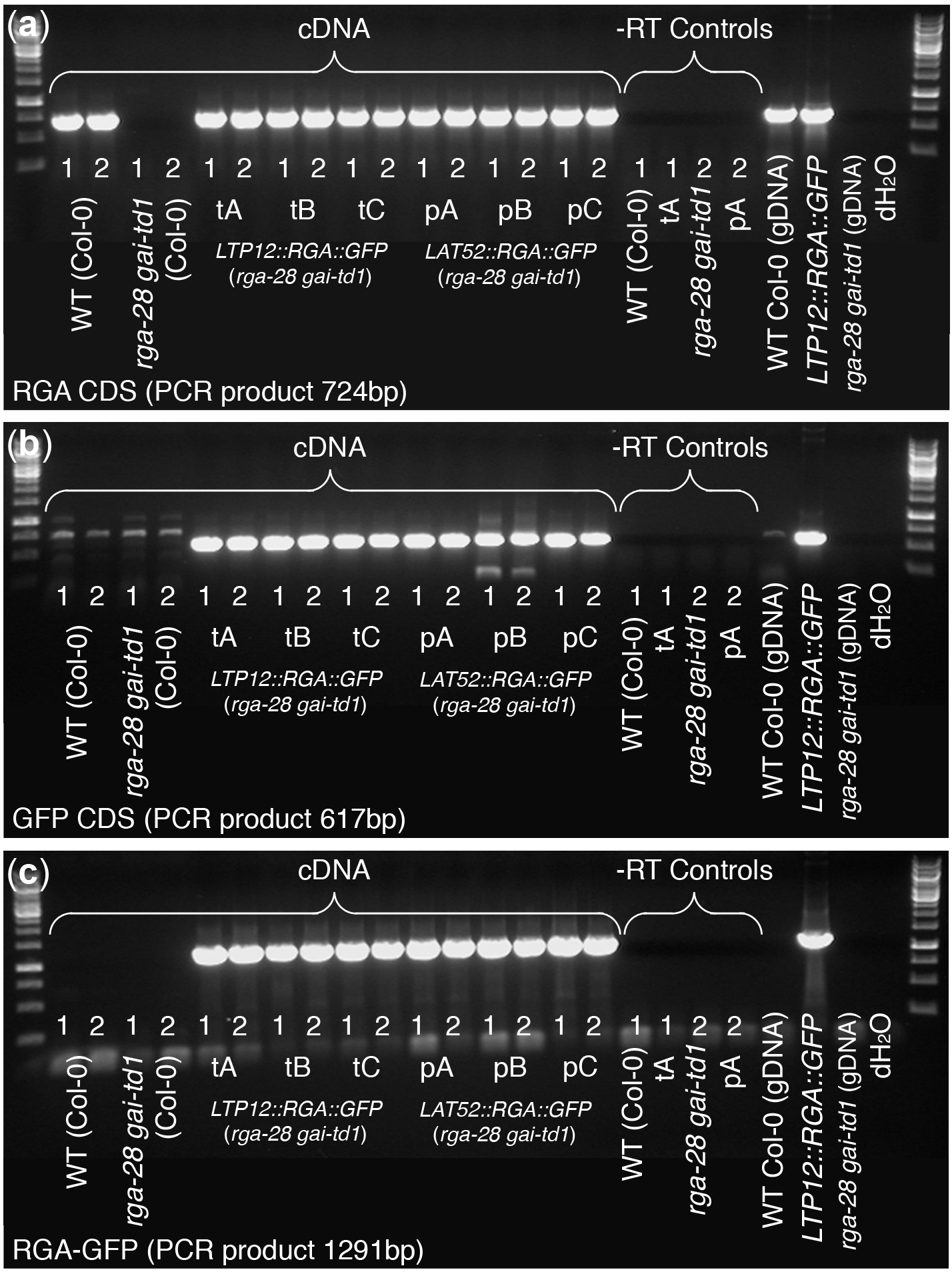


**Fig. S7.** RT-PCR analysis of RGA-GFP expression in *LTP12::RGA::GFP* and *LAT52::RGA::GFP* transgenic lines.

(a)-(c) RT-PCR analysis of RGA-GFP fusion transcript expression in *LTP12::RGA::GFP* and *LAT52::RGA::GFP* transgenic lines, amplifying products specific for the *RGA* (a) and *GFP* (b) coding sequences, and the *RGA-GFP* junction (c). Numbered lanes indicate individual plants from each genotype or independent transgenic line tested. tA-tC and pA-pC denote separate T_0_ transformation pots (see Materials & Methods). Expected PCR product sizes are supplied in the figure.


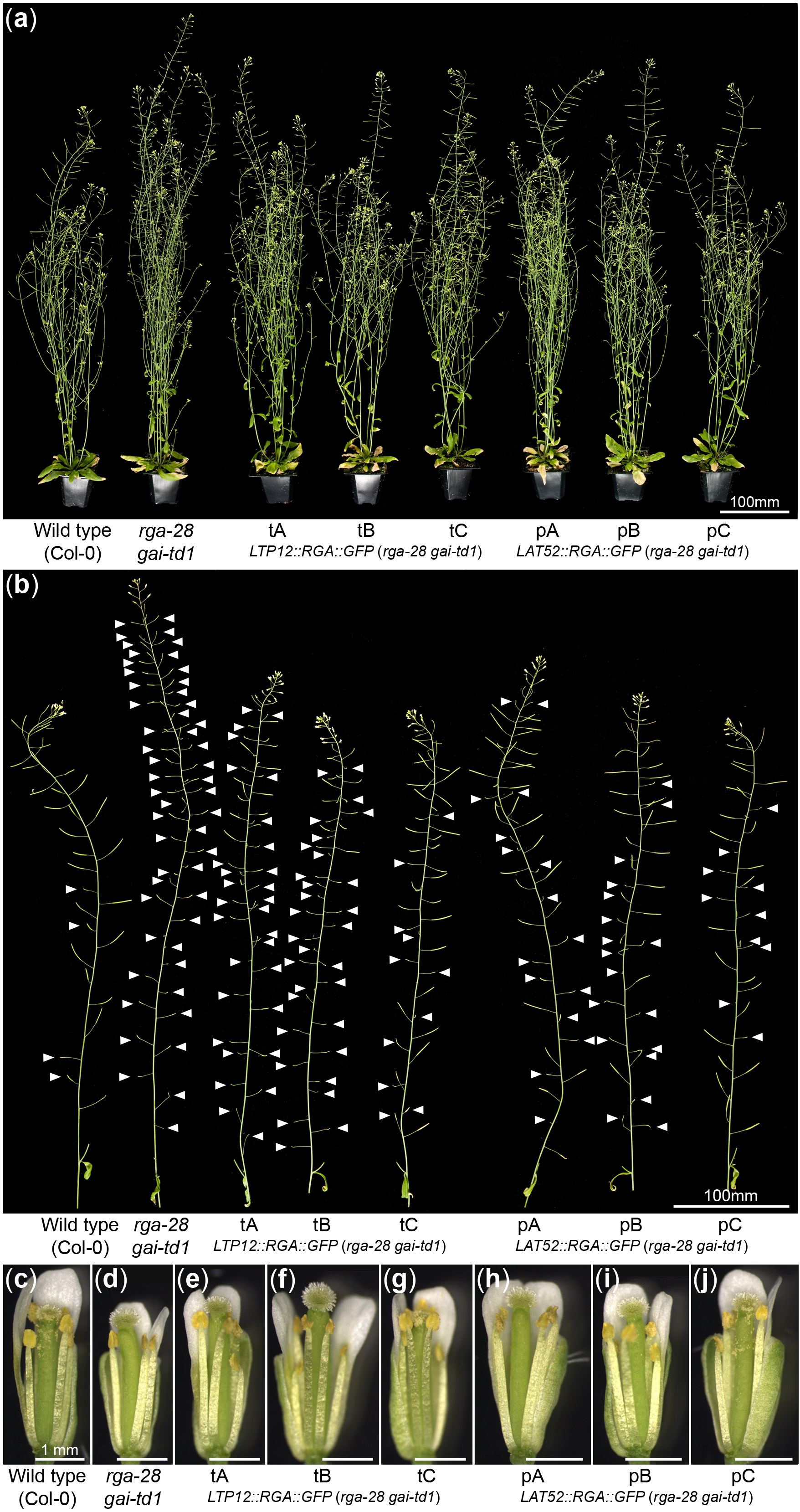


**Fig. S8.** Vegetative and reproductive phenotypes of *LTP12::RGA::GFP* and *LAT52::RGA::GFP* transgenic lines.

(a),(b) Whole plant (a) and primary inflorescence (b) phenotypes of *LTP12::RGA::GFP* and *LAT52::RGA::GFP* T_3_ homozygous lines compared against wild type Col-0 and the *rga-28 gai-td1* (Col-0) background.

Plants are shown at 39 days old. tA-tC and pA-pC denote separate T_0_ transformation pots (see Materials & Methods). White arrows indicate infertile silique positions.

(c)-(j) Floral phenotypes of the lines described in (a) and (b).


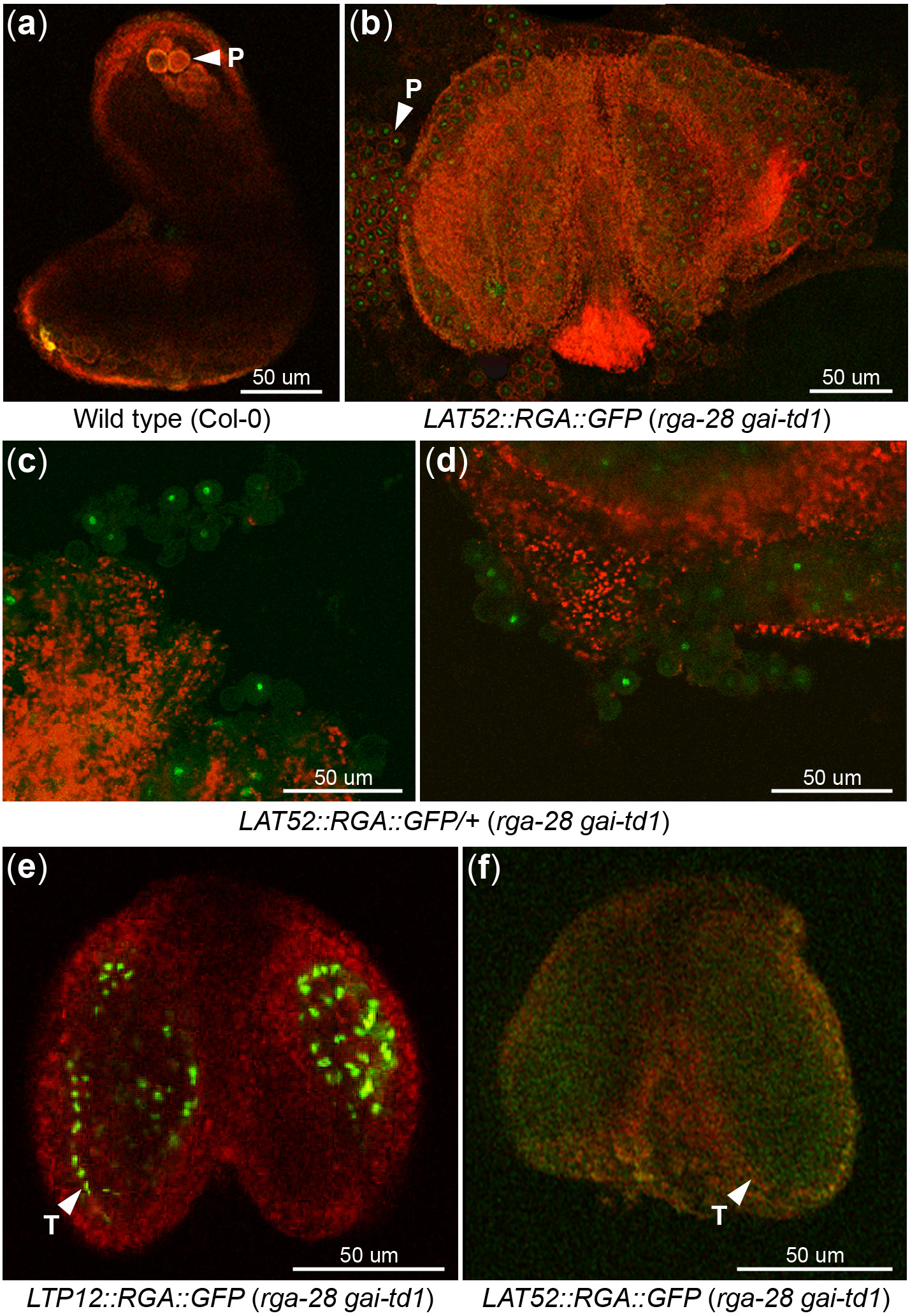


**Fig. S9.** Fluorescence analysis of *LTP12::RGA::GFP* and *LAT52::RGA::GFP* expression.

(a),(b) GFP fluorescence of wild-type Col-0 (a) and transgenic T_3_ *LAT52::RGA::GFP* (b) anthers, taken under the same exposure (see Materials & Methods). GFP fluorescence is visible in the pollen nuclei of *LAT52::RGA::GFP*, but not in wild type Col-0.

P, pollen.

(c),(d) GFP fluorescence segregating in developing pollen of anthers from two hemizygous T_2_ LAT52::RGA::GFP/+ (*rga-28 gai-td1*) individuals, taken from independent transgenic lines.


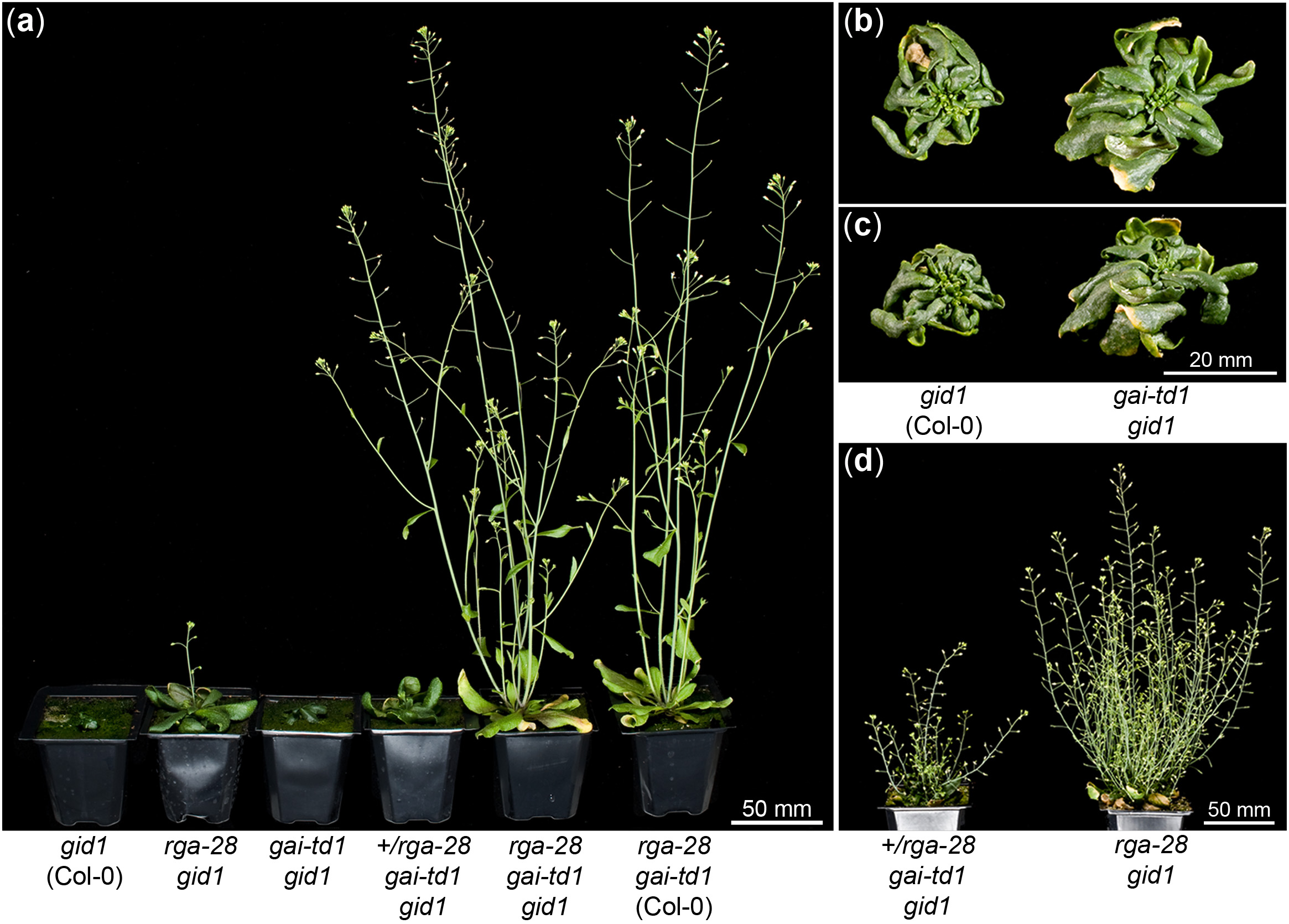


**Fig. S10** Genetic analysis of *rga gai gid1* mutant phenotypes.

(a) Vegetative phenotypes of *rga-28 gai*-td1 combinatorial mutants in the *gid1a-1 gid1b-1 gid1c-1* (*gid1*) triple GA-insensitive background, as specified. Plants are shown at 33 days old.

(b),(c) Comparison of *gid1a-1 gid1b-1 gid1c-1* (*gid1*) triple and *gai-td1 gid1* quadruple mutant vegetative phenotypes.

(d) Comparison of the *rga-28 gid1* quadruple and *+/rga-28 gid1* mutant phenotypes, demonstrating a dosage effect of RGA on plant stature.

Plants shown in (b)-(c) and (d) are 50 and 48 days old, respectively

**Table S1** PCR primer sequences.

(a)

| **Locus** | **Allele** | **Primer** | **Sequence** |
| --- | --- | --- | --- |
| ***RGA*** | WT | RGAgenoF | CGATTGTCCAACCACGGG |
|  |  | RGAR_201 | CAGCTAAGCATCCGATTTGC |
|  | *rga-28* | rga28-244 | ATGGCGGAGGTTGCTTTGAAACTCGAACA |
|  |  | LB(rga-28) | GGCAATCAGCTGTTGCCCGTCTCACTGGTG |
|  | *rga-30* | SALK_137951_RP | TACCGTGGATTCGTTGCTAAC |
|  |  | LBa1 | TGGTTCACGTAGTGGGCCATCG |
| ***GAI*** | WT | GAI_TDNA_LP3 | CGGTAACGGCATGGATGAG |
|  |  | GAI_TDNA_RP | AGCTTCGGCGAAGTAAGTAGC |
|  | *gai-td1* | LB3 | TAGCATCTGAATTTCATAACCAATCTCGATACAC |
|  |  | GAI_TDNA_RP | (As above) |
|  | *gai-td2* | SK14663_RP | TTAACCGGAAAACAGGAGGAC |
|  |  | pSKTAIL_L2 | TGGACGTGAATGTAGACACGTCG |
|  | *gai-td3* | SK20878_RP | CATTTAGCTGAGGCGATTCAC |
|  |  | pSKTAIL_L2 | (As above) |

**(b)**

| **T-DNA** | **Primer** | **Sequence** |
| --- | --- | --- |
| ***LTP12::RGA*** | pGWBF | CCTCTTCGCTATTACGCCAGC |
|  | LTP12seqR | CTTATGTTATTCTTGGGTACATGTCAAAC |
| ***LAT52::RGA*** | pGWBF | (As above) |
|  | LAT52seqR | GAAGTGATTGTAGCAATTGACTCGG |
| **RGA CDS** | RGAgenoF | (As above) |
|  | RGAR_201 | (As above) |
| **RGA-GFP** | RGAseqF | CAAATCGGATGCTTAGCTGTGTC |
|  | GFP-R2 | GCACACCGTAAGCGAAAGTAGTAAC |
| **GFP** | GFP-F | CTGTCAGTGGAGAGGGTGAAGG |
|  | GFP-R | CATGCCATGTGTAATCCCAGC |

**(c)**

| **Fragment** | **Primer** | **Sequence** |
| --- | --- | --- |
| ***Xba*I-LTP12-*Xba*I** | LTP12promXbaF | CTCTAGACATGCACTAGATCGATTTACG |
|  | LTP12promXbaR | CTCTAGAGCTTTTACTTTGTTGAGGTCG |
| ***Xba*I-LAT52-*Xba*I** | LAT52promXbaF | CTCTAGAGTCGACATACTCGACTCAGAAGGTATTG |
|  | LAT52promXbaR | CTCTAGATAATTGGAATTTTTTTTTTTGGTGTGTGTAC |
| ***Sal*I-RGA (Δstop)-*Not*I** | rga17SalF | CGTCGACATGAAGAGAGATCATCACCAATTCC |
|  | rga17NotR | CGCGGCCGCATGTACGCCGCCGTCGAGAG |

(a) Primer sequences used in genotyping of the *rga-28* and *gai-td1* mutant alleles.

(b) Primer sequences used in genotyping and RT-PCR analysis of *LTP12::RGA::GFP* and *LAT52::RGA::GFP* transgenic lines.

(c) Primers used in amplification of the *LTP12* and *LAT52* promoter fragments (see Materials and Methods) and the *RGA* CDS.

**Table S2** Col-0 and L*er* respond differently to chemical and genetic GA overdose.

| **Genotype** | **Flowering Time (Total Leaves)** | **Primary Inflorescence Height (mm)** | | **Number of Vegetative Internodes (V.I.)** | | **Mean V.I. Length (mm)** | | **Silique Length**  **(mm)** | |
| --- | --- | --- | --- | --- | --- | --- | --- | --- | --- |
|  |  | -GA | +GA | -GA | +GA | -GA | +GA | -GA | +GA |
| **WT**  **(Col-0)** | 14.417 | 403.5 | 526.2* | 2.500 | 4.167* | 28.60 [1.4503] | 31.60 [1.4956] | 15.30 [1.1840] | 12.93* [1.1043] |
| ***35S::***  ***GA20ox1* (Col-0)** | 13.333**^a^** | 485.6**^a^** | 597.8**^c^*** | 4.250**^a^** | 4.250 | 32.33 [1.5028] | 39.63**^c^*** [1.5939] | 13.81**^a^** [1.1373] | 11.96* [1.0667] |
| ***ga2ox***  **(Col-0)** | 14.333 | 461.6**^a^** | 521.9* | 3.580**^b^** | 4.417* | 26.88 [1.4237] | 32.63 [1.5093] | 13.70**^a^** [1.1312] | 11.66**^d^*** [1.0588] |
| ***rga-28***  ***gai-td1* (Col-0)** | 13.792 | 453.9**^a^** | 571.7c* | 3.500**^b^** | 4.500* | 22.15**^a^** [1.3413] | 33.24* [1.5158] | 4.29**^b^** [0.6281] | 6.51**^e^*** [0.8119] |
| **WT**  **(L*er*)** | 12.500**^b^** | 277.9**^b^** | 289.9**^d^** | 3.000 | 4.083* | 36.92**^b^** [1.5329] | 32.60 [1.5085] | 12.75**^a^** [1.1053] | 11.18**^d^*** [1.0479] |
| ***rga-24***  ***gai-t6***  **(L*er*)** | 10.991**^c^** | 295.5**^b^** | 338.1**^e^*** | 2.917 | 2.997**^c^** | 29.40**'** [1.4608] | 35.89 [1.5507] | 9.61**^c^** [0.9759] | 8.78**^f^** [0.9411] |
| *1% LSD* | ***0.6327***  *121 d.f.* | ***36.15 (40.92)*** *119 (63) d.f.* | | ***0.5954 (0.6093)*** *121 (103) d.f.* | | ***[0.06842] ([0.09006])***  *121 (36) d.f.* | | ***[0.04383] ([0.0502])*** *121 (60) d.f.* | |

Values shown are means (flowering time, *n* = 24; silique length, *n* = 36; other characters, *n* = 12; see Materials & Methods). Transformed values are given in square brackets. Rounded brackets indicate LSDs and d.f. for comparing means between GA treatments. Superscript letters indicate genotypes significantly different from the Col-0 wild-type within that GA treatment, with different letters denoting genotypes that are significantly different from one another. Where not otherwise indicated, significant difference between *rga-24 gai-t6* and L*er* wild type is marked by an apostrophe. Asterisks denote a significant effect of GA treatment.

**Table S3** Modelling the effect of GA treatment on floral organ growth in wild-type Col-0.

| **Col-0 GA Treatment** | **Pistil length**  **y = *exp*(*A* + *B*x + *C*x^2^)** | | | **Stamen length**  **y = *D* + (*E*/(1 + *exp*(-*F*(x - *G*))))** | | | | **Anther length**  **y = (*H* + *J*x)*exp*(-*K*x)** | | |
| --- | --- | --- | --- | --- | --- | --- | --- | --- | --- | --- |
|  | ***A*** | ***B*** | ***C*** | ***D*** | ***E*** | ***F*** | ***G*** | ***H*** | ***J*** | ***K*** |
| **-GA** | 0.8970 | 0.03180 | 0.0003480 | 0.8250 | 2.430 | 0.2490 | -5.60 | 0.39480 | 0.004950 | 0.022970 |
| **+GA** | 0.7940 | 0.02330**^*^** | 0.0002150 | 0.8170 | 1.890 | 0.2250 | -6.80 | 0.34440**^*^** | 0.005450 | 0.028590**^*^** |
| *5% LSD*  *(18 d.f.)* | ***0.1264*** | ***0.00544*** | ***0.0001349*** | ***0.0996*** | ***1.046*** | ***0.1109*** | ***6.47*** | ***0.01902*** | ***0.000606*** | ***0.003863*** |

See Method S1 for a detailed description of the statistical modelling used. Individual model parameters are defined as capital letters in the relevant equation included in each table section. Parameter estimates shown are means of plant-specific values (*n* = 10). Models account for a mean percentage variance across the plant-specific data (R^2^) of 94.96% (pistil), 94.68% (stamen) and 70.40% (anther), respectively. Comparisons between control (-GA) and GA-treated (+GA) growth conditions were made using 5% LSDs (as shown). Asterisks denote a significant difference between GA conditions for an individual parameter.

**Table S4** Expression of GA biosynthetic and signalling genes during wild-type L*er* pollen development.

| **Gene Name** | **Locus** | **Normalised Expression Values** | | | |
| --- | --- | --- | --- | --- | --- |
|  |  | **Unicellular Microspore** | **Bicellular Pollen** | **Tricellular Pollen** | **Mature Pollen** |
| **CPS** | At4g02780 | 0 | 0 | 0 | 0 |
| **KO** | At5g25900 | 219.6835 | 234.1135 | 0 | 0 |
| **KAO** | At1g05160 | 180.0770 | 191.8200 | 0 | 0 |
| **GA20ox1** | At4g25420 | 482.8050 | 496.3815 | 0 | 0 |
| **GA20ox2** | At5g51810 | 0 | 200.9155 | 184.5365 | 133.553 |
| **GA20ox3** | At5g07200 | 0 | 0 | 0 | 0 |
| **GA20ox4** | At1g60970 | 50.8875 | 44.4981 | 0 | 0 |
| **GA20ox5** | At1g44090 | 0 | 0 | 0 | 0 |
| **GA3ox1** | At1g15530 | 0 | 0 | 0 | 0 |
| **GA3ox2** | At1g80340 | 0 | 0 | 0 | 0 |
| **GA3ox3** | At4g21690 | 231.4905 | 286.4595 | 153.2820 | 161.6270 |
| **GA3ox4** | At1g80330 | 0 | 0 | 0 | 0 |
| **GID1a** | At3g05120 | 0 | 0 | 0 | 0 |
| **GID1b** | At3g63010 | 0 | 0 | 0 | 0 |
| **GID1c** | At5g27320 | 0 | 0 | 0 | 0 |
| **RGA** | At2g01570 | 173.9565 | 195.586 | 0 | 0 |
| **GAI** | At1g14920 | 179.5980 | 212.0425 | 0 | 0 |
| **RGL1** | At1g66350 | 72.36925 | 0 | 0 | 0 |
| **RGL2** | At3g03450 | 97.94625 | 120.9990 | 0 | 0 |
| **RGL3** | At5g17490 | 0 | 0 | 0 | 0 |
| **MYB33** | At5g06100 | 205.6235 | 250.6950 | 146.0335 | 130.6690 |
| **MYB65** | At3g11440 | 243.1870 | 250.9270 | 340.1920 | 0 |
| **SLEEPY** | At4g24210 | 222.2620 | 212.3065 | 0 | 0 |
| **SNEEZY** | At5g48170 | 96.90275 | 100.52835 | 143.6130 | 0 |

Data reproduced from Honys & Twell (2004), with permission. Expression data is from the L*er* ecotype. An expression value of ‘0’ indicates that expression of a particular gene was not above detection threshold in all technical replicates.

**Method S1**

Supporting experimental procedures - nonlinear modelling of floral organ growth.

Floral organ growth under control and GA-treated conditions was modelled from a population of 20 plants (*n* = 10), with inflorescence development synchronised by harvesting at the opening of the 10^th^ flower. To capture floral organ growth during flower opening, individual organ lengths were recorded from 8 floral buds preceding and 4 following the 10^th^ open flower in each floral cluster. Different total numbers of floral buds were found in floral clusters under control and GA-treated conditions (p < 0.05, t-test), so a relative developmental scale between inflorescences was calculated using the mean time interval between individual buds of each inflorescence (total buds divided by the time from first bud appearance on the inflorescence to harvesting). Nonlinear models describing the growth of reproductive organs across relative development time (h) were fitted to individual primary inflorescences, performing a regression for each plant as a first stage of this analysis. As a second stage, significance of differences between genotypes was assessed at the population level by ANOVA of the plant-specific estimates of parameters contained within the models (Table S2). The nonlinear models (exponential for pistil, logistic for stamen and critical exponential for anther growth) were chosen to best describe the growth of each organ across the specified range of development [Causton DR, Venus JC. 1981. *The biometry of plant growth*. London, UK: Edward Arnold (Publishers) Ltd.].
